# Layer-Dependent Interaction Effects in the Electronic Structure of Twisted Bilayer Graphene Devices

**DOI:** 10.1021/acs.nanolett.3c00253

**Published:** 2023-07-24

**Authors:** Nicholas Dale, M. Iqbal Bakti Utama, Dongkyu Lee, Nicolas Leconte, Sihan Zhao, Kyunghoon Lee, Takashi Taniguchi, Kenji Watanabe, Chris Jozwiak, Aaron Bostwick, Eli Rotenberg, Roland J. Koch, Jeil Jung, Feng Wang, Alessandra Lanzara

**Affiliations:** † Department of Physics, University of California, Berkeley, California 94720, United States; ‡ Materials Sciences Division, Lawrence Berkeley National Laboratory, Berkeley, California 94720, United States; § Department of Materials Science and Engineering, University of California at Berkeley, Berkeley, California 94720, United States; ∥ Department of Physics, University of Seoul, Seoul, 02504, Korea; ⊥ Department of Smart Cities, University of Seoul, Seoul, 02504, Korea; # Interdisciplinary Center for Quantum Information, Zhejiang Province Key Laboratory of Quantum Technology and Device, State Key Laboratory of Silicon Materials, and School of Physics, Zhejiang University, Hangzhou 310027, China; ¶ International Center for Materials Nanoarchitectonics, National Institute for Materials Science, 1-1 Namiki, Tsukuba 305-0044, Japan; ○ Research Center for Functional Materials, National Institute for Materials Science, 1-1 Namiki, Tsukuba 305-0044, Japan; ▽ Advanced Light Source, Lawrence Berkeley National Laboratory, Berkeley, California 94720, United States; ◆ Kavli Energy NanoScience Institute at University of California Berkeley and Lawrence Berkeley National Laboratory, Berkeley, California 94720, United States

**Keywords:** twisted bilayer graphene, moiré heterostructures, ARPES, symmetry-breaking, electron−electron interaction, band gap.

## Abstract

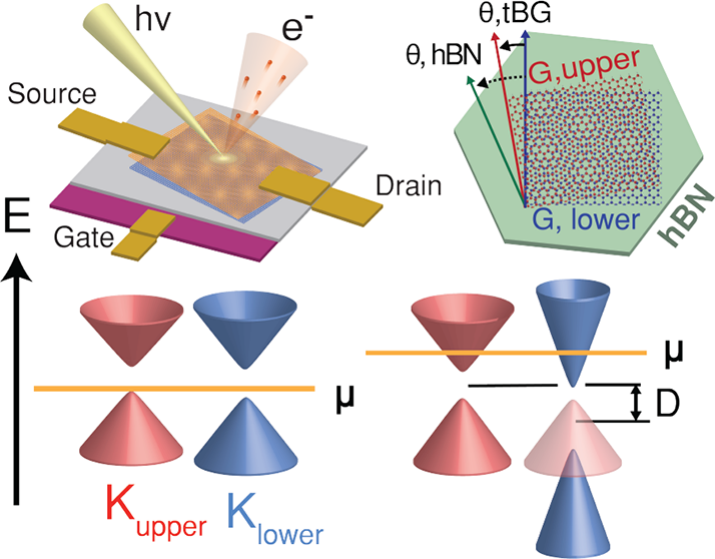

Near the magic angle, strong correlations drive many intriguing phases in twisted bilayer graphene (tBG) including unconventional superconductivity and chern insulation. Whether correlations can tune symmetry breaking phases in tBG at intermediate (≳ 2°) twist angles remains an open fundamental question. Here, using ARPES, we study the effects of many-body interactions and displacement field on the band structure of tBG devices at an intermediate (3°) twist angle. We observe a layer- and doping-dependent renormalization of bands at the *K* points that is qualitatively consistent with moiré models of the Hartree–Fock interaction. We provide evidence of correlation-enhanced inversion symmetry-breaking, manifested by gaps at the Dirac points that are tunable with doping. These results suggest that electronic interactions play a significant role in the physics of tBG even at intermediate twist angles and present a new pathway toward engineering band structure and symmetry-breaking phases in moiré heterostructures.

The search for intriguing phases of matter often treads along common avenues: generating strong correlations and breaking symmetries in materials. Correlations develop when the ratio of interaction strength *U* to bandwidth *W* in a system becomes large (*U*/*W* ≫ 1), presenting instabilities to myriad ground states. Twisted bilayer graphene is an especially popular host of correlated phases^1−3^ due to the ability to tune the bandwidth with extreme precision using the twist angle,^4,5^ and the ability to tune the interaction strength *U* in situ through charge carrier^6^ and substrate-based^7^ screening.

Most studies of renormalization effects in twisted bilayer graphene to date have focused on how the overall bandwidth is modified by interactions. Indeed, several theoretical works predict significant band structure modification from the Hartree^8−11^ and Hartree–Fock interaction,^12^ which have been shown in some cases to drive correlated phases outside the twist angle regime predicted by early band structure models.^6,12,13^ However, graphene devices are rarely inversion symmetric,^14^ especially in the presence of a displacement field, and at twist angles θ ≳ 2° the *K* point electronic structure is layer polarized,^15^ in principle enabling each layer to host differing electronic properties or phases. When inversion symmetry is broken, so far it is unclear how introducing correlations affect electronic structure in each individual layer of twisted graphene and whether these differences could independently tune symmetry breaking phases in each layer. Here, we use gated ARPES^16−18^ to directly measure the layer-dependent electronic structure of an intermediate angle (3°) twisted bilayer graphene device as a function of electrostatic doping and displacement field. The high momentum and energy resolution of our experiment allow us to identify direct evidence for layer-dependent interaction-driven band narrowing as well as a substrate-induced band gap at the *K* points. We find that a combination of displacement field and electron–electron interactions is able to enhance this band gap within just a single layer and thus tune the level of symmetry breaking in the system with atomic layer precision.

Figure 1a,b presents the sample configuration and geometry, respectively, used for the gated ARPES experiment, and panel c shows the scanning electron micrograph of the sample. The sample was fabricated using the tear-and-stack flip chip method described in ref (19) to minimize surface roughness, enabling high momentum resolution for photoemission experiments. False color identifies regions of the top monolayer graphene (orange), the bottom monolayer graphene (blue), hBN (white), and graphite back gate (purple). Using a 1 μm beam spot via a capillary focusing optic,^20^ we produce a scanning photoemission micrograph (SPEM) (panel d) that maps the real space configuration of the graphene photoelectrons at *E*_F_ and confirms the sample geometry. The intensity of the signal deriving from graphene scales with the layer number:^19^ the middle region has a stronger intensity than the region on the left, matching the expected location of the tBLG and monolayer graphene regions, respectively as outlined in panel b.

**Figure 1:**
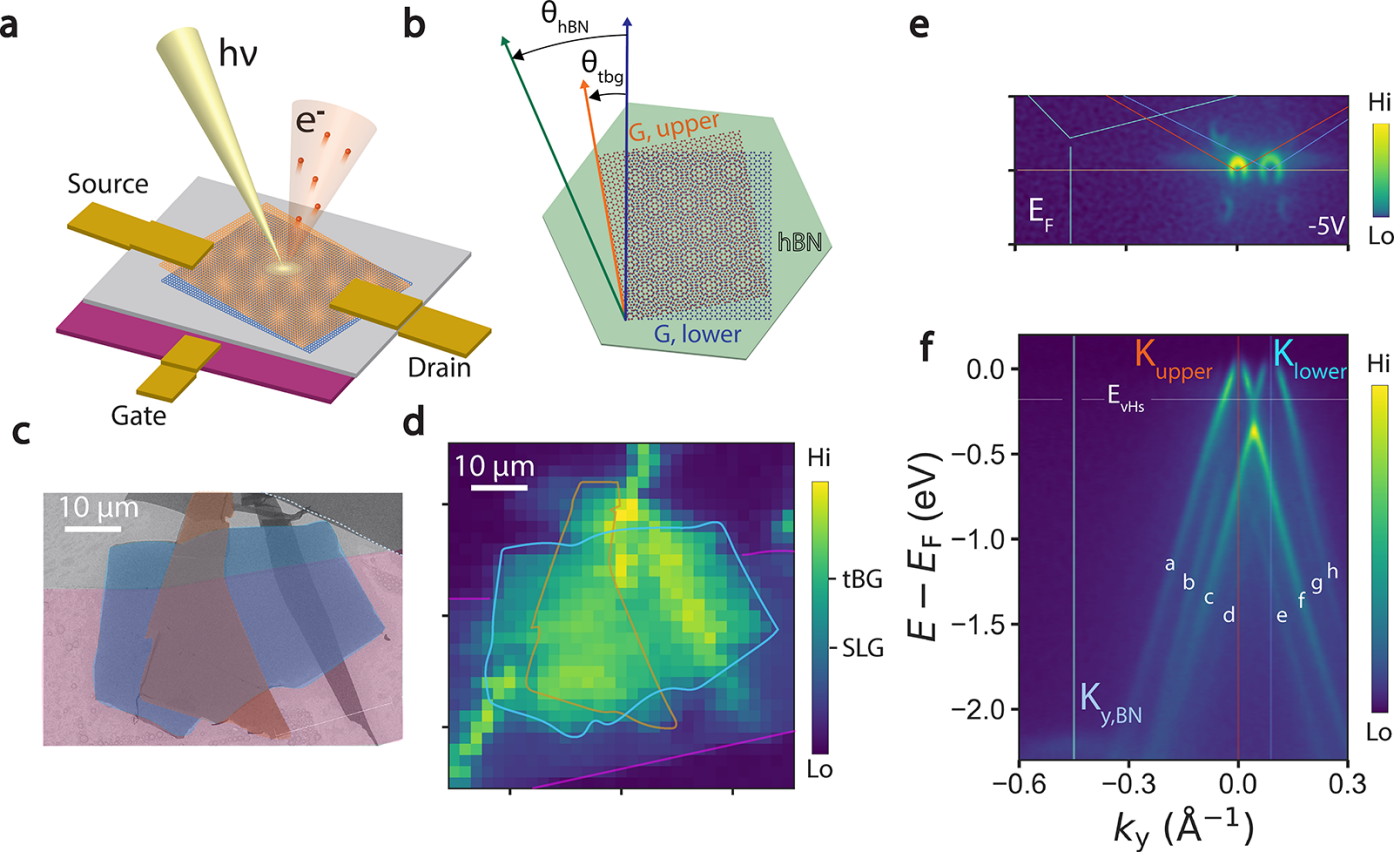
Gated ARPES on twisted bilayer graphene (a–d) schematic of experimental setup (a) angular geometry (b) of the twisted graphene/hBN sample. (c, d) scanning electron micrograph (c) and scanning photoemission microscopy (SPEM) image integrated over states at *E*_*F*_ (d). Regions of graphene upper layer (orange), lower layer (blue), hBN (gray), and graphite (purple) are outlined/filled in with false color. (e) ARPES constant energy spectrum at *E*_F_ for sample gate voltage of −5 V. Brillouin zones for the individual graphene upper (lower) layers are outlined in red (blue), while the Brillouin zone for hBN is outlined in cyan. (f) ARPES spectrum along the *K*_upper_ – *K*_lower_ direction (indicated by yellow line in part e) of the twisted graphene/hBN sample.

Figure 1e presents the ARPES constant energy map for a tBLG region at a back gate voltage of −5 V. Encompassing the *K* points of the two graphene layers are circular Fermi contours whose matrix elements smoothly drop to zero along one side, suggesting that the low energy Fermions in the sample are of Dirac nature.^21,22^ Upper and lower graphene layers can be distinguished by the difference in spectral weight near the respective *K* points, as the photoelectrons from the bottom layer are attenuated as they pass through the top layer.^23−25^ The twist angle can be measured in ARPES from the momentum separation of the *K* points of the two graphene layers using the relationship Δ*K* = 2 |*K*| sin θ/2. On the sample a twist angle of 3° was measured, corresponding to a moiré wavelength of 6 nm and a momentum separation Δ*K* ≈ 0.09 *Å*^–1^. The angular alignment of the hBN substrate can be extracted in a similar manner: the momentum separation of ≈0.54 *Å*^–1^ between the *k*_*y*_ location of the hBN *K* point (parabola at *E*_F_ – 2.2 eV in panel f) and the lower graphene *K* point indicates a ≈19° relative twist.

Figure 1f presents ARPES spectra along the *K*_upper_ – *K*_lower_ direction. At large (>2°) twist angles, the *K* point spectra for the upper and lower graphene layers can be approximated as Dirac cones.^23,25,26^ This is apparent visually by eye within ≈200 meV of the Fermi level in panel f, whose bands from left to right we label as a, c, f, and h. Around 200 meV below *E*_F_ (panel f), a van Hove singularity forms from the hybridization between intersecting bands from the lower and upper cones.^23−25^ Other evidence of graphene–graphene interlayer coupling in our sample is very prominent: Dirac cone replicas of the upper and lower layers are clearly observed at the corresponding mini Brillouin zone (mBz) corners (panel e), and at energies beyond 500 meV (panel f) a series of additional bands (b, d, e, and g) are present, similar to previous reports on SiC-supported graphene samples at similar twist angles.^26,27^

Upon applying a negative (positive) back gate voltage to the sample, the two graphene layers become doped by holes (electrons), resulting in an upward (downward) shift of the Dirac cone spectrum with respect to *E*_F_. Due to the imperfect out-of plane screening in graphene multilayers, application of a back gate voltage *V*_bg_ to our sample generates an electric field between the upper and lower graphene layers, resulting in both a difference in charge carrier density *δ n* = *n*_u_ – *n*_l_ and chemical potential difference *D* = μ_*u*_ – μ_*l*_^23,28,29^ (see Figure 2a).

**Figure 2:**
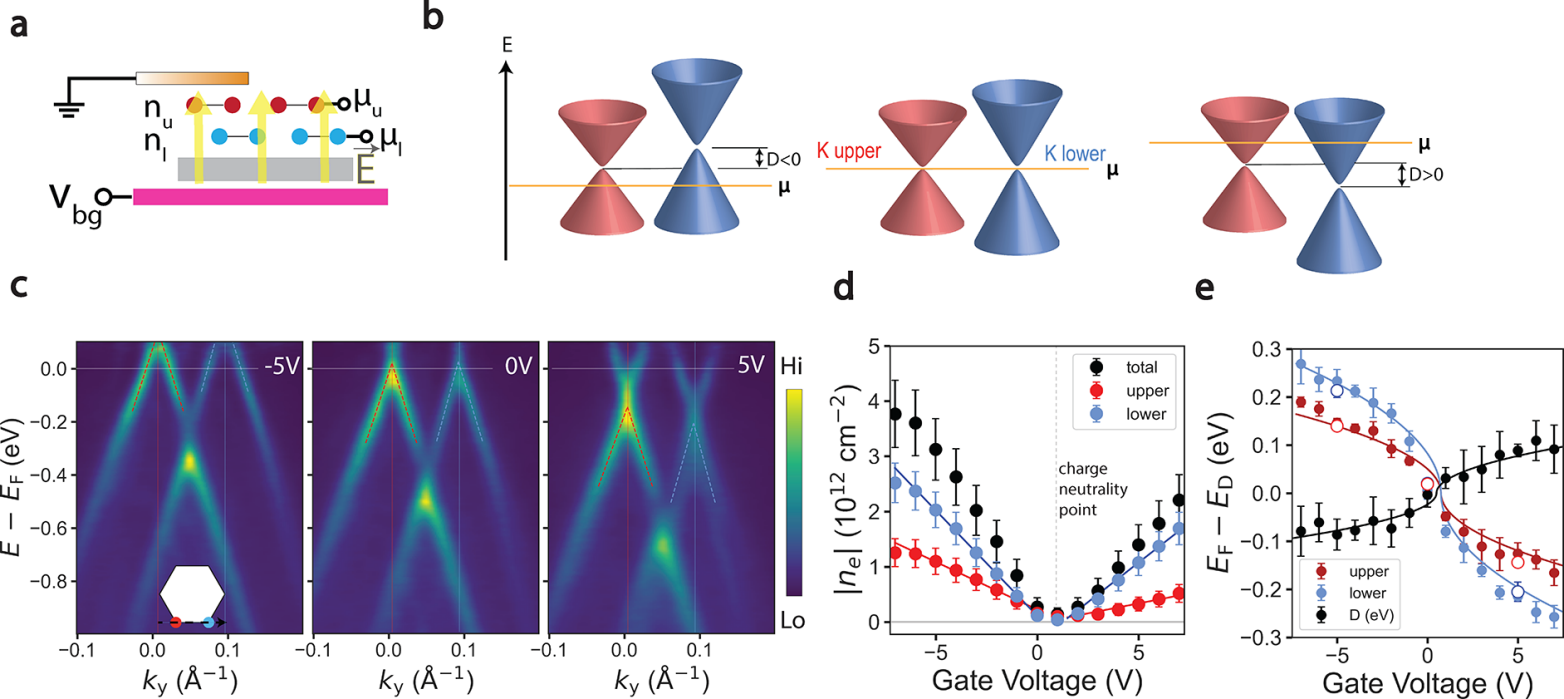
Displacement field characterization of tw-BLG device. (a) schematic of displacement field effect produced in twisted graphene bilayers under a single back gate potential. (b) Cartoons above indicate relative energy of upper (red) and lower (blue) Dirac cones upon p doping (left) at neutrality (middle) and n doping (right). (c) ARPES spectra along the *K*_upper_ – *K*_lower_ direction at (e) p doping (−5 V, left) equilibrium (0 V, middle), and n doping (5 V, right). Red (blue) dashed lines indicate linear fits to quasiparticle peak positions extracted from Lorentzian fits. (d) Carrier density *n*_e_ as a function of gate voltage, extracted from Fermi wavevector of each cone. Red (blue) curves are linear fits to the data in the upper (lower) Dirac cone away from the neutrality point. (e) *E*_*F*_ – *E*_*D*_ as a function of gate voltage, extracted from linear fits to the graphene spectra for the upper (red) and lower (blue) graphene layers. Band displacement (black) is extracted as the energy difference between the two layers. Error bars indicate statistical errors to the linear fit. Red, blue, and black curves indicate  fits to the data.

Such an effect, illustrated by the cartoon in Figure 2b, is clearly reflected in our data (Figure 2c). The lower layer receives higher absolute doping and therefore a larger absolute shift of its Dirac cone spectrum as compared to the upper layer, resulting in a band displacement D that is tunable in magnitude and sign with the back gate voltage.^23,28,29^

The carrier density, calculated as *n* = *k*_F_^2^/π for the upper and lower Dirac cones, is quantified in Figure 2d (see Supplementary Note 1 for details). Away from the neutrality point at *V*_g_ = 1 V, the density scales linearly with gate voltage, i.e., *n*(*V*_g_) ∼ *C V*_g_, implying that the system is effectively a parallel plate capacitor with geometric capacitance *C*. Near the neutrality point, *n*(*V*_g_) flattens, suggesting contributions of quantum capacitance due to a strong drop in the density of states, likely from the presence of a gap in the dispersion (see Supplementary Note 6 for more details). We will return to this point later in the text.

Linear fits to the band dispersions (dashed lines in panel c) at low energy yield the position of the Dirac point for the upper and lower layers, which are plotted as a function of gate voltage in panel e. The Dirac point position as a function of voltage roughly scale as  (red and blue curves) with *a* = 0.09 ± 0.01 and *a* = 0.06 ± 0.01 for lower and upper layers, respectively). The band displacement, taken as the difference between the two Dirac cone energies, therefore has the same qualitative scaling  where *b* = 0.03 ± 0.01.

The stronger scalings of both *E*_F_ – *E*_D_ and *n*(*V*_g_) with gate voltage in the lower layer indicates the larger amount of charge induced by the gate in the lower layer, confirming previous reports on large twist angle bilayer graphene.^23,28,29^

As we shall demonstrate in the following, both doping and displacement fields can be used to control the band structure beyond what would be expected by a single particle picture.

Figure 3 presents the evolution of the upper and lower *K* point electronic structures as a function of doping for the 3° twisted graphene sample. Figure 3b displays band dispersions along Γ – *K*_lower_ for dopings of −2.0 × 10^12^ cm^–2^, −0.1 × 10^12^ cm^–2^, and 1.0 × 10^12^ cm^–2^ (see Supplementary Note 1 for details on the calculation of the carrier density). These dispersions have been shifted by the position of the Dirac point of the upper layer for the ease of comparison. From the raw data, it is clear by eye that all three spectra are linear within 300 meV of the Dirac point. Notably, the hole-doped dispersion (green) is less steep than the dispersion at neutrality (brown), which is less steep than the dispersion at electron doping (red). Indeed, a similar effect occurs in the upper layer (Figure 3a) though at much smaller magnitude.

**Figure 3:**
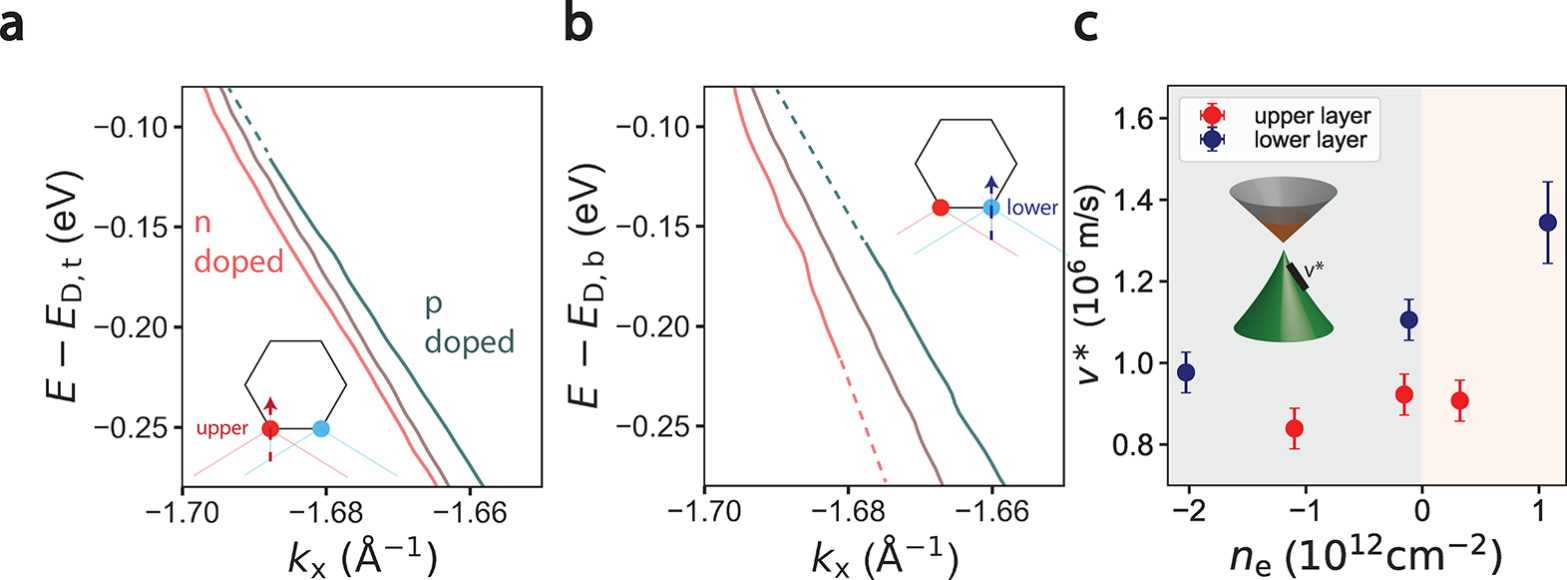
Doping-induced band renormalization. (a, b) Band dispersions as a function of doping along Γ – *K*_upper_ (a) and Γ – *K*_lower_ (b), see inset cartoon for details on the cut in momentum space. (c) Band velocities as a function of doping, for both layers, measured along energy region indicated by inset cartoon. Error bars correspond to 1σ standard deviation in the fits.

These results clearly indicate a narrowing of the valence band upon hole-doping and are summarized in panel c, where the band velocities, measured by the slope of the dispersion, are plotted as a function of doping. These results do not match the behavior of monolayer graphene,^18,30,31^ which has a logarithmic divergence of band velocity at the neutrality point. Instead, we see a near monotonic decrease in band velocity with hole-doping. Indeed, this may be explained in models of twisted bilayer graphene that incorporates the interactions spurring from change in charge distribution in the moiré unit cell.^6,8−11^ At neutrality, charge localizes on the AA sites, creating a Hartree–Fock potential that is stronger in these regions than the rest of the moiré unit cell which has more delocalized electrons. As the doping changes, charge redistributes in the moiré cell, causing band renormalization with the same doping dependence as our results: upon hole-doping, the valence band narrows, and upon electron doping, the valence band steepens.^6^ The relatively small band velocity change with doping in the upper graphene layer suggests that the electron–electron interaction is more strongly screened, perhaps due to the presence of the doped graphene layer beneath it. Indeed, reduced *v*_F_(32−35) and changes with doping^31,35,36^ are observed in single layer graphene upon increasing the dielectric constant of the substrate. The lower graphene layer, which is supported by a lower dielectric strength substrate, therefore, receives a stronger band velocity enhancement from the Hartree–Fock interaction. While the changes of band velocity with doping predicted by Hartree–Fock models are on the ≈1% scale for a doping change of ≈ *n*_*s*_/8,^6,12^ we are able to modify the band velocity by up to 40% in the lower graphene layer. Indeed these theoretical models of graphene may underestimate renormalization effects from the long-range electron–electron interaction, similar to the case of single layer graphene.^36−38^ Band structure renormalization from Hartree interaction is in fact enhanced when the initial graphene band structure incorporates the band velocity enhancements present in graphene on a dielectric substrate (see Supplementary Note 2 for more details). Further investigation is necessary to determine the exact origin of this behavior.

The difference in band velocity in the upper and lower layers is clear evidence of *C*_2*x*_ inversion symmetry breaking in the graphene sample. While this often occurs in graphene samples supported by hBN substrates^39−42^ or undergoing heterostrain,^43^ the level of symmetry breaking here is doping dependent. This signature is in other parts of the electronic structure.

Figure 4 presents the layer-dependent evolution of the *K* point electronic structure with doping and a displacement field. Charge neutral ARPES spectra at the upper layer *K* point (Figure 4a_1_) exhibit the typical Dirac cone dispersion with a crossing at the Fermi level. Given a sample temperature of 300 K, division by the Fermi Dirac distribution provides insight into the electronic structure within 4*k*_*B*_*T* ≈ 100 meV of the Fermi level (see Materials and Methods in the Supporting Information), which presents a drop in the density of states at the neutrality point followed by the bottom of a conduction band which is energetically separated from the valence band by ≈130 meV. This can be confirmed by the raw MDCs spectra (panel b_1_) which are dispersionless in a similar range in energy. Similar electronic structure is observed in the lower layer *K* point electronic structure (panels d_1_, e_1_) These data constitute direct evidence for a band gap in this twisted graphene/hBN sample near the charge neutrality point. Such property is observed in samples with broken *C*_2*z*_ inversion symmetry, which can occur in the presence of an hBN substrate.^14,39,40,42,44^

**Figure 4:**
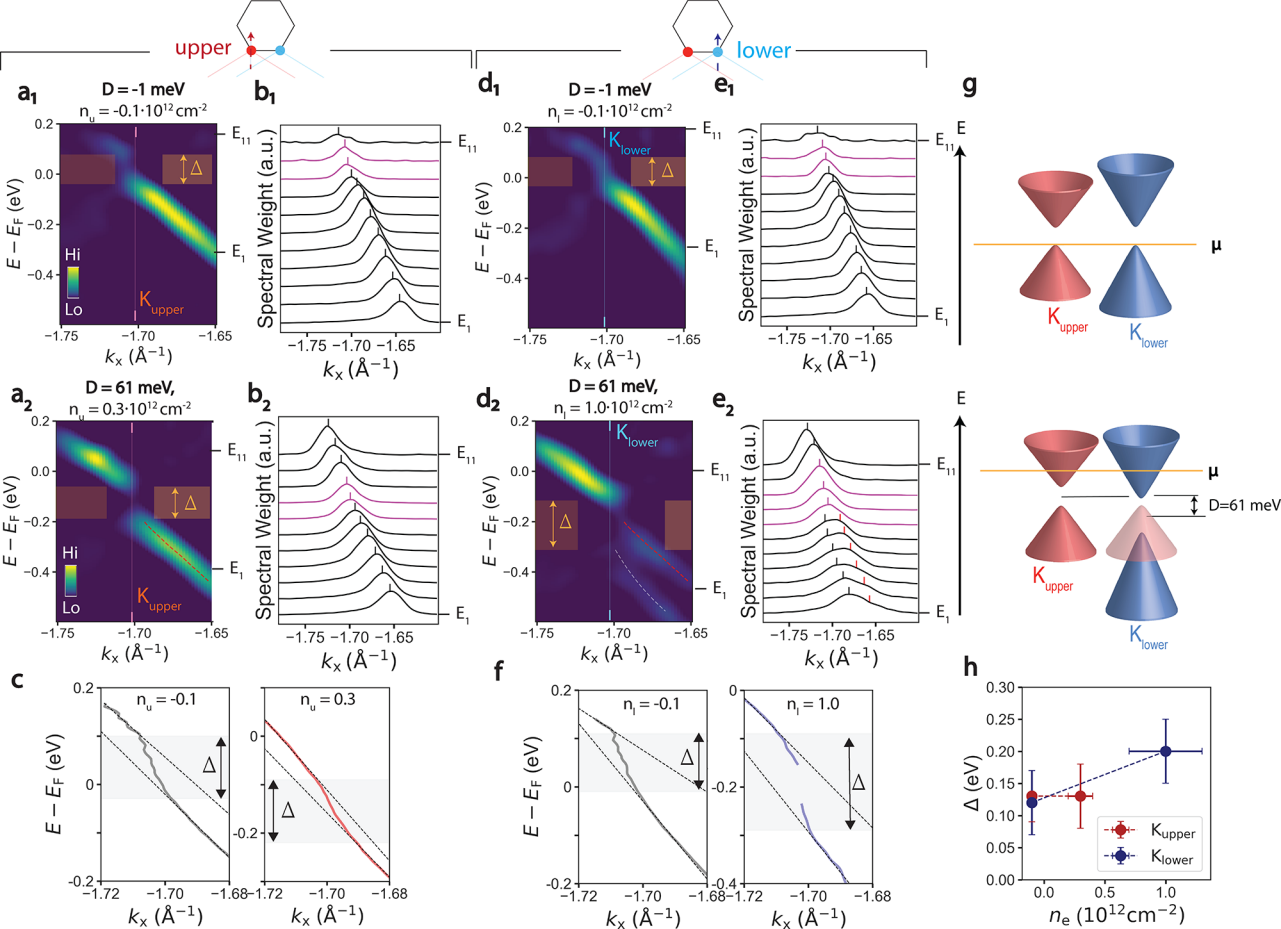
Gate-tunable gap at neutrality point. (a_1_, a_2_) 3° twisted graphene second derivative spectra along Γ – *K*_upper_ for two different displacement field values: −1 meV (a_1_) and 61 meV (a_2_). Gaps in the dispersion are indicated by orange shaded regions. Red dashed lines in the right panel are valence band dispersions deriving from the upper graphene layer. (b_1_, b_2_) Corresponding MDCs for parts a_1_ and a_2_, between energy *E*_1_ and *E*_11_. Black ticks indicate quasiparticle peak positions extracted from fitting to Lorentzian lineshapes. Purple curves are in the gap, where the peaks are dispersionless. (c) Extracted dispersions from raw data associated with parts a_1_ (left) and a_2_ (right). Dashed lines indicate linear fits to the conduction and valence dispersions. Gray regions indicate gaps in the band structure, bordered by kinks in the MDCs dispersion. (d_1_, d_2_) 3° twisted graphene second derivative spectra along Γ – *K*_lower_ for two different displacement field values: −1 meV (a_1_) and 61 meV (a_2_). Gaps in the dispersion are indicated by orange shaded regions. Red (white) dashed lines in right panel are valence band dispersions deriving from the upper (lower) graphene layer. (e_1_, e_2_.) Corresponding MDCs for parts d_1_ and d_2_, between energy *E*_1_ and *E*_11_. Black ticks indicate quasiparticle peak positions extracted from fitting to Lorentzian lineshapes. Purple curves are in the gap, where the peaks are dispersionless. Red ticks indicate quasiparticle peaks deriving from the upper graphene layer. (f) Extracted dispersions from raw data associated with d_1_ (left) and d_2_ (right). Dashed lines indicate linear fits to the conduction and valence dispersions. Gray regions indicate gaps in the band structure, bordered by kinks in the MDCs dispersion. (g) Schematic of band gap renormalization with displacement field and doping. (h) Summary of band gaps in the upper and lower layers as a function of doping.

Upon electron doping the sample, the upper layer electronic structure (panels a_2_, b_2_) appears similar to the spectrum at neutrality but rigidly shifted by about 150 meV, with the band gap remaining relatively constant. However, the lower layer band structure (panels d_2_, e_2_) undergoes significant modification: whereas the spectrum at neutrality has one valence band, the electron doped spectrum (panel d_2_) exhibits two. Indeed, the raw MDCs spectra (panel e_2_) exhibit 2 peaks, represented as shoulders, between 500 and 200 meV below *E*_F_. Interestingly, these peaks at higher momenta have dispersion and energy very similar to those of the valence band of the upper Dirac cone (red curves in panel a_2_ and red peaks in panel b_2_). We therefore propose the lower energy band (red dashed lines in panel d_2_) to be a replica of the upper layer electronic structure and to be independent of the lower layer electronic structure. Indeed, such replicas manifest in zone-unfolded band structure calculations in the presence of an electric field between upper and lower graphene layers (see Supplementary Note 3 for more details). The lower layer valence band at higher energy (white dashed line in panel d_2_) is therefore dramatically separated from the conduction band, indicating a significant increase in the band gap size with doping and displacement.

The layer and doping dependent behavior of the band gaps is confirmed by the quasiparticle dispersions in the upper and lower *K* points (Figure 4, parts c and f) respectively. Gaps in ARPES manifest as kinks or regions of abrupt upturn in the MDCs dispersions,^42,45,46^ and in Dirac materials such as graphene these regions are bounded by linear dispersions.^46^ In both layers at all dopings, there is a nonzero region of energy at which the dispersions have an abrupt upturn bounded by linearly dispersive features. This behavior is consistent as a function of momentum surrounding the upper and lower *K* points, demonstrating the unambiguous signature of a band gap (see Supplementary Note 4 for more details). The magnitude of the band gap is determined from the energetic distance between linearly dispersive regions in the band structure which are denoted by dashed black lines. Upon application of a 61 meV displacement and doping the upper layer by 0.3 × 10^12^ cm^–2^, the bandgap at *K*_upper_ remains constant at 140 ± 50 meV. However, upon applying the same band displacement and doping the lower layer by 1.0 × 10^12^ cm^–2^ the bandgap at *K*_lower_ increases from 130 ± 50 meV to 200 ± 50 meV. Such layer-dependent doping behavior of band gaps in our data, summarized in panel h, are qualitatively consistent with the amount of inversion symmetry-breaking present in the sample.

The layer and doping dependent behavior of the band gaps is confirmed by the quasiparticle dispersions in the upper and lower *K* points, (Figure 4, parts c and f) respectively. Gaps in ARPES manifest as kinks or regions of abrupt upturn in the MDCs dispersions,^42,45,46^ and in Dirac materials such as graphene these regions are bounded by linear dispersions.^46^ In both layers at all dopings, there is a nonzero region of energy at which the dispersions have an abrupt upturn bound by linearly dispersive features. This behavior is consistent as a function of momentum surrounding the upper and lower *K* points, demonstrating the unambiguous signature of a band gap (see Supplementary Note 4 for more details). The magnitude of the band gap is determined from the energetic distance between linearly dispersive regions in the band structure, which are denoted by dashed black lines. Upon application of a 61 meV displacement and doping the upper layer by 0.3 × 10^12^ cm^–2^, the bandgap at *K*_upper_ remains constant at 140 ± 50 meV. However, upon applying the same band displacement and doping the lower layer by 1.0 × 10^12^ cm^–2^ the bandgap at *K*_lower_ increases from 130 ± 50 meV to 200 ± 50 meV. Such layer-dependent doping behavior of band gaps in our data, summarized in panel h, are qualitatively consistent with the amount of inversion symmetry-breaking present in the sample. Indeed, the inversion symmetry-breaking produced by a misaligned hBN substrate^41,44^ can be significantly enhanced by electron–electron interactions.^7,41,47^ In graphene, the magnitude of interaction-driven gap enhancement scales with the interaction strength,^7,41,47^ which in tBG scales linearly with doping.^8−11^ Therefore, a charge imbalance between the two graphene layers, generated, e.g., via a displacement field, can drive a doping-dependent gap mechanism which enables layer-dependent gap enhancements such as those seen in our experiment.

The magnitude of gaps observed in our data is much larger than those observed in the literature at similar twist angle.^48−50^ Whereas other probes such as transport and STS commonly define gaps as regions with zero density of states, ARPES defines the band gap using the band edges, at which the density of states rarely has an abrupt drop to zero.^42^ Impurities may also contribute to in-gap states that decrease the apparent gap size in a transport or STS measurement. Notably, both single layer^51^ and bilayer graphene^52^ in particular exhibit in-gap conductive resonances as a response to the presence of charged impurities, which significantly alters the accuracy of a gap measurement using STS d*I*/d*V* or transport. ARPES can still measure the gap in these cases because band edges are bordered by regions of dispersionless spectral weight.^45^ Finally, as transport is only sensitive to the spatially integrated electronic states at the Fermi level, it may miss the gap entirely because (1) at the neutrality point, the spatially inhomogeneuos doping present in tBG samples^6,53,54^ can produce insulating regions adjacent to conductive regions, which upon spatial integration could appear as a minor decrease in conductivity^48,50^ (see Supplementary Note 5 for more details), and (2) upon electron doping, the gap increase observed here occurs when the Dirac point is below the Fermi level.

The data reported here provide evidence for a method of tuning the band velocities and band gaps in twisted bilayer graphene in operando. While we considered several alternative explanations (see Supplementary Note 7), we believe that these effects can be best explained by a combination of the substrate interaction and the spatially inhomogeneous Hartree–Fock interaction, which is controlled within different layers by using a displacement field. Indeed, these interactions can qualitatively explain the presence of the gap at charge neutrality, the linear dependence of band velocity with doping and the layer-dependent gap enhancement upon electron doping the sample.

In conclusion, we have demonstrated that gated ARPES is an exceptional tool to study the interplay of interactions and symmetry breaking in 2D homo- and heterostructures. Our results indicate that both the spatially inhomogeneous Hartree interaction and a displacement field can be used to independently tune the bandwidth and band gaps in twisted bilayer graphene, opening up the intriguing possibility to engineer bands and enabling access to novel correlated phases in a larger range of twisted homobilayers^6,55,56^ and other heterostructures.^57−60^

## Supporting Information Available

Methods for tBG device fabrication, ARPES measurements and data analysis; notes on carrier density measurements, Hartree interaction effects in 3° tBG, evidence for presence of replicas of primary bands in 3° tBG, and additional analysis of the gap at the K_upper_ and K_lower_ points in the band structure; and dditional notes on the effects of spatial inhomogeneity on the carrier density measurements, as well as a discussion of the origin of doping- and layer-dependent band velocity band gap enhancements (PDF)
