# Supplementary material for: Layer-Dependent Interaction Effects in the Electronic Structure of Twisted Bilayer Graphene Devices
Source: Nano Lett. 2023 Jul 24;23(15):6799–806. doi: 10.1021/acs.nanolett.3c00253 (PMC10424631; doi:10.1021/acs.nanolett.3c00253)
Supplement: Supplementary file 1 — nl3c00253_si_001.pdf [file nl3c00253_si_001.pdf]

Layer-dependent interaction effects on the electronic structure of twisted bilayer graphene devices

Nicholas Dale,[1,2] M. Iqbal Bakti Utama,[2,3] Dongkyu Lee,[4,5] Nicolas Leconte,[4] Sihan Zhao,[6] Kyunghoon Lee,[1,2] Takashi Taniguchi,[7] Kenji Watanabe,[8] Chris Jozwiak,[9] Aaron Bostwick,[9] Eli Rotenberg,[9] Roland J. Koch,[9] Jeil Jung,[4,5] Feng Wang,[1,2,10] and Alessandra Lanzara[1,2,10]

[1]*Department of Physics, University of California, Berkeley, CA, 94720, USA*

[2]*Materials Sciences Division, Lawrence Berkeley National Laboratory, Berkeley, CA, 94720, USA*

[3]*Department of Materials Science and Engineering, University of California at Berkeley, Berkeley, CA, 94720, USA*

[4]*Department of Physics, University of Seoul, Seoul, 02504, Korea*

[5]*Department of Smart Cities, University of Seoul, Seoul, 02504, Korea*

[6]*Interdisciplinary Center for Quantum Information, Zhejiang Province Key Laboratory of Quantum Technology and Device, State Key Laboratory of Silicon Materials, and School of Physics, Zhejiang University, Hangzhou 310027, China*

[7]*International Center for Materials Nanoarchitectonics, National Institute for Materials Science, 1-1 Namiki, Tsukuba 305-0044, Japan*

[8]*Research Center for Functional Materials, National Institute for Materials Science, 1-1 Namiki, Tsukuba 305-0044, Japan*

[9]*Advanced Light Source, Lawrence Berkeley National Laboratory, Berkeley, CA, 94720, USA*

[10]*Kavli Energy NanoSciences Institute at University of California Berkeley and Lawrence Berkeley National Laboratory, Berkeley, CA, 94720, USA*

(Dated: April 10, 2023)

CONTENTS

I. Materials and Methods 3
1. Sample Preparation: 3
2. ARPES Measurements and Analysis: 3

II. Supplementary Notes 4
1. Carrier Density Measurements 4
2. Hartree Interaction Effects in 3° tBG 5
3. Replicas of Primary Bands in 3° tBG 7
4. Gap Analysis at the K Point 11
5. Twist Angle Inhomogeneity Effects on Electronic Structure 15
6. Spatial Inhomogeneity Effects on Carrier Density Measurements 17
7. Discussion of the Origin of Doping- and Layer- Dependent Band Velocity and Band Gap Enhancements 21

III. Supplementary References 23

References 23

I. MATERIALS AND METHODS

1. Sample Preparation:

Flakes of single-layer Graphene and hexagonal Boron Nitride were exfoliated onto Silicon Wafers with 90nm-thick oxide. The sample was constructed using a method similar to that used in [1]. A stamp comprised of Polypropylene carbonate (PPC), and Polydimethylsiloxane (PDMS), and transparent tape was used to pick up Graphite, hBN, and Graphene in sequential order. The PPC stamp holding the stack was flipped onto a 90nm oxidized Si wafer with the Graphene facing up, and the polymer was subsequently removed by annealing in a vacuum furnace at 350C for 10 hours. Contacts were patterned onto each sample surface using electron-beam lithography followed by evaporation of 5nm Cr and 50nm Au.

2. ARPES Measurements and Analysis:

Samples were measured using a Scienta R4000 Hemispherical Analyzer at the nanoARPES branch of beamline 7.0.2 (MAESTRO) at the Advanced Light Source using a photon energy of 74 eV, a temperature of 300K,and a pressure better than 1e-10 Torr. The beam was capillary refocused [2] to a spot size of $\sim 1\mu\mathrm{m}\,\mathrm{x}\,1\mu\mathrm{m}$. The overall energy and momentum resolution was 30meV and $0.014\mathring{A}^{-1}$, respectively. The sample was doped electrostatically using a Keithley 2450 Source Meter.

All ARPES data in this paper were analyzed using pyARPES, an open-source python-based analysis framework[3]. Spectra presented in the figures have been deconvolved by the experimental energy and momentum resolution using the Lucy-Richardson method as described in ref [4]. Spectra presented in Figs 2-4 have additionally been divided by the Fermi-Dirac distribution following the methods in ref[4]. Second derivative spectra have been smoothed by 10 meV and $0.007\mathring{A}^{-1}$ which is smaller than the energy and momentum resolution of the experiment.

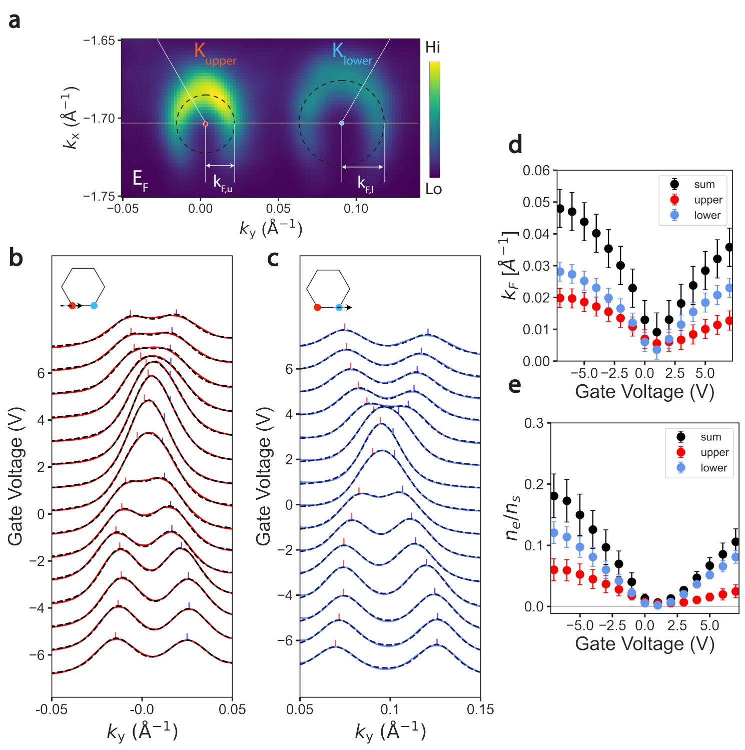

Supplementary Figure 1: (**a.**) 3° twisted graphene Fermi surface at a gate voltage of -5V. White lines indicate Fermi wavevector k_{F} measured as the radius of the Fermi surface for both upper and lower layers.(**b.**,**c.**) MDCs at E_{F} as a function of applied gate voltage for upper (**b-**) and lower (**c-**) layers along momentum direction indicated by inset cartoon. Black dashed lines are fits to the spectra based on Voigt lineshapes, peaks positions labelled with red and blue markers. (**d.**) Fermi wavevector k_{F} as a function of gate voltage for both layers. Error bars are estimated from the experimental momentum resolution and the broadness of the MDC. (**e.**) Carrier density n_{e} as a function of gate voltage for both layers, calculated using Luttinger's theorem: $n_{\mathrm{e}} = {k_{\mathrm{F}}}^2/\pi$, and normalized by the superlattice filling n_{s}, whose formula is given in the text below. Error bars represent errors propagated from estimates of k_{F}.

II. SUPPLEMENTARY NOTES

1. Carrier Density Measurements

The charge-carrier density can be obtained from the size of the Fermi surface using Luttinger's theorem. For each graphene layer, the Fermi surface (Fig 1a) is a circle with

a radius of the Fermi wavevector k_{F}, i.e. $n_{\mathrm{e}} = k_{\mathrm{F}}^2/\pi$. We extract k_{F} as half the distance between spectral peaks in the MDCs at the Fermi level, which are displayed in Fig 1b,c for the upper and lower layers of the 3° twisted graphene, respectively. The summary of extracted k_{F} values, and calculated n_{e} values are presented in Fig 1d and Fig 1e, respectively. Here we normalize the doping to the total filling of the moiré unit cell, which at small twist angles is approximated as $n_{\mathrm{s}} = 4\nu \approx 10^4 \frac{8*\theta^2}{a^2\sqrt{3}}$, where θ is the twist angle and a is the graphene lattice constant of $2.46\mathring{A}$[5]. Error bars for k_{F} are estimated based on the broadness of the bands and the momentum resolution, which was $\sim 0.014\,\mathring{A}^{-1}$, while error bars for n_{e} are obtained by propagating errors in k_{F}.

As mentioned in the main text, the bottom layers of each sample consistently receives larger doping than does the upper layers due to the inability to screen the field produced by a back gate voltage[6, 7]. Away from neutrality, both samples appear to have a roughly linear dependence between doping and gate voltage i.e. $n_{\mathrm{e}} \simeq CV_{\mathrm{g}}$, which is expected when treating the system as a parallel-plate capacitor with geometric capacitance C. At the neutrality point, a dip in geometric capacitance indicates the presence of a small gap, perhaps from the inversion symmetry broken by the hBN substrate [8] (see Supplementary Note 6 for more details).

Due to the relatively large twist angle of the samples measured here, the overall doping range is not very large with respect to the filling required to occupy 4 electrons per moiré unit cell. The presence of trace amounts of PPC, slightly hole dopes the sample, leaving the neutrality point around 1V applied back gate voltage[9].

2. Hartree Interaction Effects in 3° tBG

The doping-induced renormalization effects presented in the main manuscript are an order of magnitude larger than what is predicted from Hartree and Hartree Fock interaction models in the literature for small twist (1.4°) angle graphene[10–12]. Here we present a Hartree model for 3 ° twisted graphene in Fig2 which incorporates the band velocity enhancement from the long range electron-electron interaction in single layer graphene[9], and exhibits band renormalization of a qualitatively comparable to that of the experiment. We then

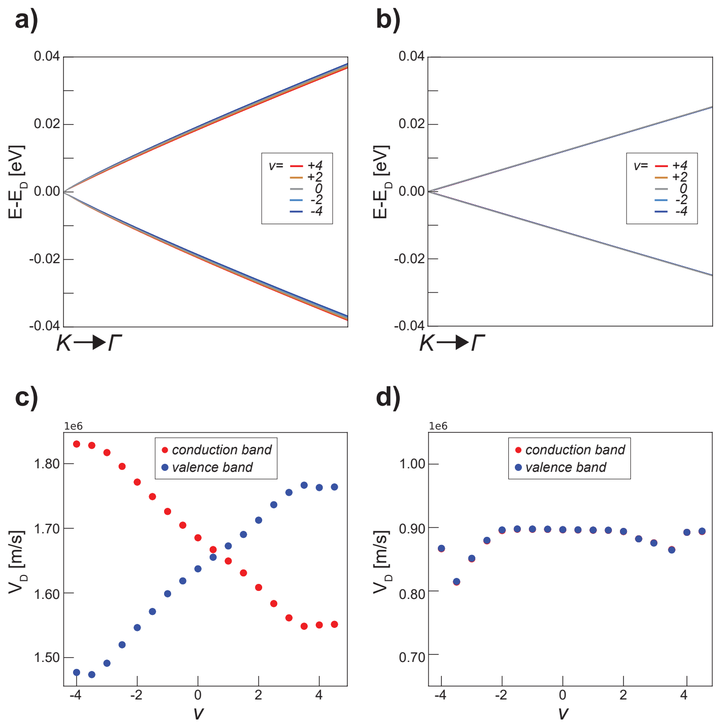

Supplementary Figure 2: Hartree Interaction Effects in 3° tBG. (**a.,b.**) 3° twisted graphene K point band structure as a function of filling for an effective exchange model of graphene which incorporate long-range electronic interactions (**a-**) and a bare Hartree model (**b-**) (**c.,d.**) Summary of Dirac point band velocities as a function of doping for band structure in **a** (**c-**), and **b** (**d-**).

compare this model to a bare Hartree model which exhibits minimal band renormalization. Below we breifly summarize the methodology behind these calculations.

The mean-field Hartree Hamiltonian we used is given in the following expression:

$$H_H = H + \sum_{i,\sigma\neq\sigma'} U\delta\rho_{i,\sigma'}c^\dagger_{i,\sigma}c_{i,\sigma} + \sum_{i,j,\sigma,\sigma'} V_{ij}\delta\rho_{i,\sigma'}c^\dagger_{j,\sigma}c_{j,\sigma} \tag{1}$$

where the first term is tight-binding Hamiltonian described in Supplementary Note 3, the second term is the on-site Hubbard Hamiltonian and the last is Hartree Hamiltonian with $V_{ij} = \frac{1}{\epsilon|r_i - r_j|}$ in a unit of Hartree. The local density fluctuation $\delta\rho_{i,\sigma} \equiv \langle n_{i,\sigma}\rangle - n^0_{i,\sigma}$ is considered to avoid double-counting of coulomb interaction where n^0_i is local neutral density. In order to check the trend of doping-induced effects, the typical values of $U = 5$ and $\epsilon = 4$ were used. The self-consistent calculation was performed on a 5×5 Monkhorst-Pack mesh.

To check the effect on the long-range exchange interaction, we defined an effective exchange model, which incorporates the long-range coulomb interaction effects present in single layer graphene on dielectric substrates near the charge neutrality point [9, 13–15]. In this model, the first order parameter t_0 of intra-layer hopping is redefined as a function of k point as

$$t_0' = t_0(1 + \frac{2.2}{4\epsilon} ln \frac{1.75}{min(|k-K|, |k-K'|)}). \quad (2)$$

Fig 2a presents the results of the effective exchange model. As the filling changes from negative to positive, a clear steepening of the band structure can be observed in the valence band, along with a corresponding softening of slope in the conduction band. The band velocity as a function of filling, measured from the slope of these dispersions, is summarized in panel c. Over the filling range explored in our experiment (approximately ν = -1 to ν = 1), the Dirac point velocity v_{D} undergoes a 5% velocity enhancement, which is comparable in magnitude to the changes seen in the experiment for the upper layer of graphene. By comparison, the bare Hartree model (panels b,d), undergoes near zero band structure modification, and therefore presents no band velocity enhancement, confirming the results of previous calculations [12, 16–18].

3. Replicas of Primary Bands in 3° tBG

The analysis here explores whether the extra set of shallow bands present near $\mathrm{K}_{\mathrm{lower}}$ originate from electronic states in the lower layer of graphene. Indeed, two valence bands at the lower K point would suggest a substantially different interpretation of the evolution of the band gap with doping in the material. In the following, we show evidence that these extra bands are replicas of electronic structure from the upper K point, as was discussed in the main text and thus rule out that the second band comes from the lower K point. Here we present layer-resolved spectral function calculations of 3° twisted graphene (panels a-c) including a 100 meV displacement field in absence of Hubbard or Hartree terms from Eq. (1) that show distinct replicas of the $K_{upper}(K')$ electronic structure within $K_{lower}(K)$, producing two Dirac cones in a single K valley. The energy at which the replica cone in one layer occurs matches exactly the energy at which the main cone appears in the other layer,

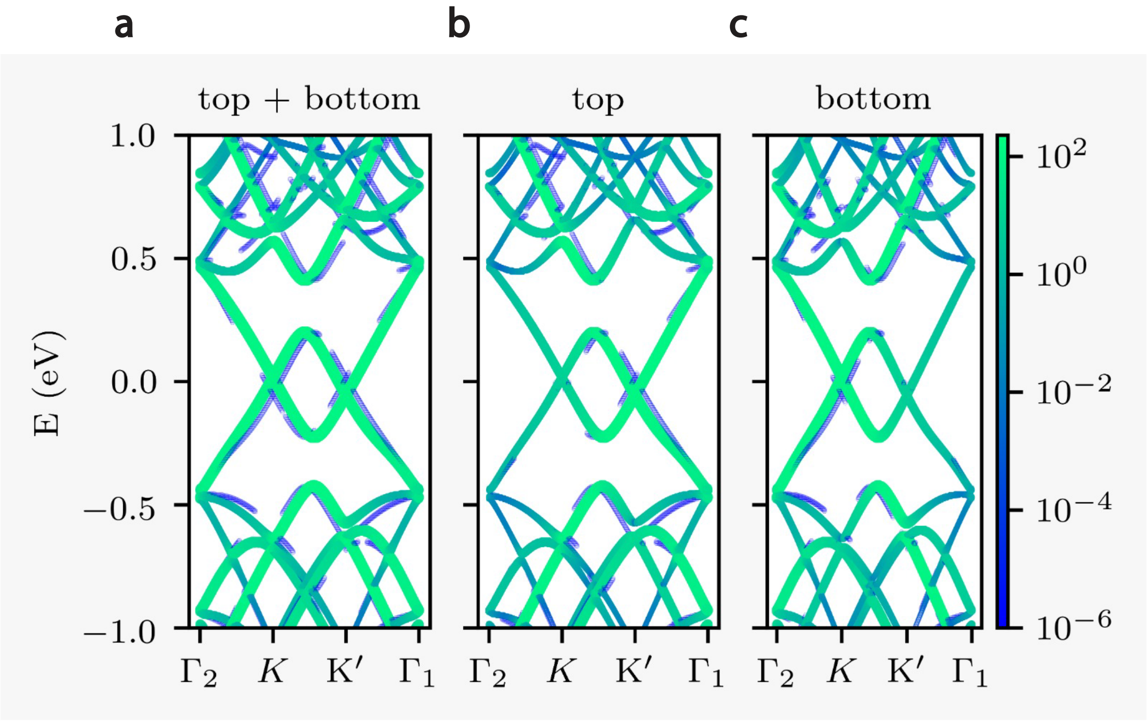

Supplementary Figure 3: Dirac Cone Replicas in the Band Structure. (**a.-c.**) spectral functions for 3° twisted graphene arising from both layers (**a-**), the top graphene layer (**b-**), and the bottom layer (**c-**) under a band displacement of 100 meV (details on calculation in the text).

suggesting they are indeed replicas of these original cones. Indeed, within each valley the distinct cones are separated by exactly 100meV, reflecting the energy separation between the upper and lower layers. As these replica cones are faint, they may be present but unreported in previous zone unfolded calculations [19].

Below we describe the methodology of the band structure calculations performed here, which are described extensively in ref [20]. The calculations use KLIFF [21]-fitted DRIP potential [22] parameters reproducing EXX-RPA level long-range interactions in bilayer graphene [23]. The energy mimization is performed using the LAMMPS package [24] using the *cg* algorithm with a timestep of 0.001 ps. The REBO2 Brenner potential [25] is used for intralayer C-C interactions.

We define the tight-binding (TB) Hamiltonian as

$$\hat{H} = \sum_{i}^{n_{at}} \epsilon_i |i\rangle\langle i| + \sum_{i,j}^{n_{at}} t_{ij} |i\rangle\langle j| \tag{3}$$

where $|i\rangle$ is a basis of localized states at site i and the eigenfunctions are given as

$$|k\rangle = \frac{1}{\sqrt{n_{at}}} \sum_{j}^{n_{at}} e^{i\boldsymbol{k}\cdot\boldsymbol{r}_j} |j\rangle \tag{4}$$

with n_{at} the number of atoms and where $\boldsymbol{k} = (k_x, k_y)$. The onsite energies ϵ_i on the one hand are defined using the F2G2 model of graphene [26] and can be modulated due to an electric field by potential energy shifts given by

$$\Delta\epsilon = \epsilon_i^{L_2} - \epsilon_i^{L_1} = \text{diag}\left(-\frac{N-1}{2}, -\frac{N-1}{2}+1, \ldots, +\frac{N-1}{2}\right) \cdot \frac{\Delta V}{N-1}\mathbb{1}. \tag{5}$$

where L_1 or L_2 indicates if an atom belongs to layer 1 or 2. On the other hand, the hopping terms t_{ij} can be separated into an intralayer t_{ij}^{intra} contribution and an interlayer t_{ij}^{inter} contribution where the former follows the previously mentioned F2G2 model of graphene [26] and the latter are using a parametrizatioin that puts the twisted bilayer graphene magic angle at 1.08° due to the specific choice of $S = 0.895$ when using the DRIP potential for interlayer interactions and the REBO2 potential for intralayer interactions during the LAMMPS minimization procedure in the following expression [27]

$$t_{ij}^{\text{inter}} = S \exp\left[\frac{c_{ij} - p}{q}\right] t_{\text{TC},ij}^{\text{inter}} \tag{6}$$

where $p = 3.25$ Å and $q = 1.34$ Å control the interlayer distance-dependent fitting of the tunneling at the K-point and

$$t_{\text{TC},\,ij} = V_{pp\pi}(r_{ij}) \left[1 - \left(\frac{c_{ij}}{r_{ij}}\right)^2\right] + V_{pp\sigma}(r_{ij}) \left(\frac{c_{ij}}{r_{ij}}\right)^2 \tag{7}$$

where

$$V_{pp\pi}(r_{ij}) = V_{pp\pi}^0 \exp\left(-\frac{r_{ij} - a_0}{r_0}\right) \tag{8}$$

and

$$V_{pp\sigma}(r_{ij}) = V_{pp\sigma}^0 \exp\left(-\frac{r_{ij} - c_0}{r_0}\right) \tag{9}$$

with the interlayer distance $c_0 = 3.35$ Å, the rigid interatomic carbon distance in graphene $a_0 = 1.42$ Å, the transfer integral between nearest-neighbor atoms $V_{pp\pi}^0 = -2.7$ eV, the

transfer integral between two vertically aligned atoms $V^0_{pp\sigma} = 0.48$ eV, the decay length of the transfer integral set to $r_0 = 0.184a$ such that the next-nearest intralayer coupling becomes 0.1 $V^0_{pp\sigma}$ and the magnitude of the interatomic distance $r_{ij} = |\boldsymbol{r}_{ij}|$. The cutoff for this distance-dependent model is set to 4.9 Å beyond which additional contributions do not affect the observables anymore [28].

For the spectral function [29–35] calculations, we use the implementation outlined in Ref. [20] based on Ref. [29] for which we remind the expressions here. In such calculations, the zone-folded large supercells that capture the moire physics can be represented in the Brillouin zone of a smaller periodic unit cell through

$$A_{\boldsymbol{k},n}(E) = \sum_{\boldsymbol{K}J} |\langle \boldsymbol{k}n|\boldsymbol{K}J\rangle|^2 \, A_{\boldsymbol{K}J,\boldsymbol{K}J}(E) \tag{10}$$

with $|\boldsymbol{K}J\rangle$ eigenbands of the supercell that are labeled with capital letters and smaller letter n labels the Bloch function basis $|\boldsymbol{k}n\rangle$ with the localized orbital n in the reference small unit cell. The latter are used to to distinguish the layer and sublattice and in our case, we chose to represent the results for $n = 1$. In the above expression $A_{\boldsymbol{K}J,\boldsymbol{K}J}(E)$ reduces to a $\delta(E - \epsilon_{\boldsymbol{K}J})$ function at the eigenvalue of the superlattice system and $\langle \boldsymbol{k}n|\boldsymbol{K}J\rangle$ is a structure factor that is modulated by a position-dependent phase term as

$$\langle \boldsymbol{k}n|\boldsymbol{K}J\rangle = \sqrt{L/l} \sum_N w_N e^{i\boldsymbol{k}\cdot\boldsymbol{R}(N)} \delta_{n,n'(N)} \delta_{[\boldsymbol{k}],\boldsymbol{K}} \langle \boldsymbol{K}N|\boldsymbol{K}J\rangle \tag{11}$$

where $[\boldsymbol{k}]$ denotes the k-point folded into the supercell BZ, where N and n are the orbital indices in the supercell and normal reference cell respectively and with $\boldsymbol{R}(N)$ giving the position of the atom N in the supercell. The last factor in this expressions are the coefficients of the supercell eigenstate $|\boldsymbol{K}J\rangle$ projected in the tight-binding basis $|\boldsymbol{K}N\rangle$, with L and l equalling the number of k-points in the supercell and reference small cell Brillouin zone (BZ) respectively. $w_N \leq 1$ is a coefficient that allows to tune the relative contribution of certain atoms to capture the top layer contribution that is usually stronger in experiment. Here we set this value to 1 as we plot the top and bottom layer contributions separately. Photon polarization effects are ignored although they could alter the momentum distribution anisotropy [36, 37].

4. Gap Analysis at the K Point

The analysis here is very sensitive to the exact locations of the K_{upper} and K_{lower} points. Indeed, given the steep linear dispersion of graphene, a small misalignment can result in an overestimate of the band gap size [8, 38, 39]. Figs 4 and 5 address the gap location with high momentum resolution ARPES spectra surrounding K_{upper} and K_{lower} points, respectively. Figs 4b1-b5 present several cuts around K_{upper} (See schematic drawing and corresponding Fermi surface in panel a) near the neutrality point. These second derivative spectra exhibit clear separation between conduction and valence bands. Following the analysis in the main text, such separation has a signature in the MDCs spectra: the intensity drops, and the peaks follow an unusally steep dispersion (purple regions in panels c1-c5). These very steep regions appear as kinks in the extracted MDCs dispersions (panels d1-d5) whose size is measured by the energies there is deviation from linearity (black dashed lines in panels d1-d5),[38]. The band separation as a function of momentum, summarized in panel i, is clearly nonzero for all cuts measured, with a minimum at the K_{upper} point. Similar findings are found for the electron doped spectra in panels e - h, where the gap size is nearly identical to that of the spectrum at neutrality for all momenta.

Fig 5 presents the same analysis applied to the lower layer Dirac cone. The second derivative spectra at both the neutrality point (panels b1-b5) and electron doping (panels f1-f5) exhibit energetic separation between valence and conduction bands, whose edges are denoted by the orange horizontal lines. As mentioned in the main text, the electron doped spectra (panels f1 -f5) show a clear signature of two valence bands, where the lower energy band is a replica of the upper cone valence band, whose dispersion is indicated by the red dashed curves. The regions where nonreplica bands have deviations from linearity in their dispersions (panels d1-d5 for charge neutrality, and h1-h5 for electron dopings), determines the band separation, which is summarized in Fig 5i. For all momenta and dopings measured, the band separation near K_{lower} is nonzero, confirming the existence of gaps in the electronic structure within the resolution of the experiment. For all momenta near K_{lower}, the band separation is larger at electron doping than at the neutrality point, confirming the results presented in the main text.

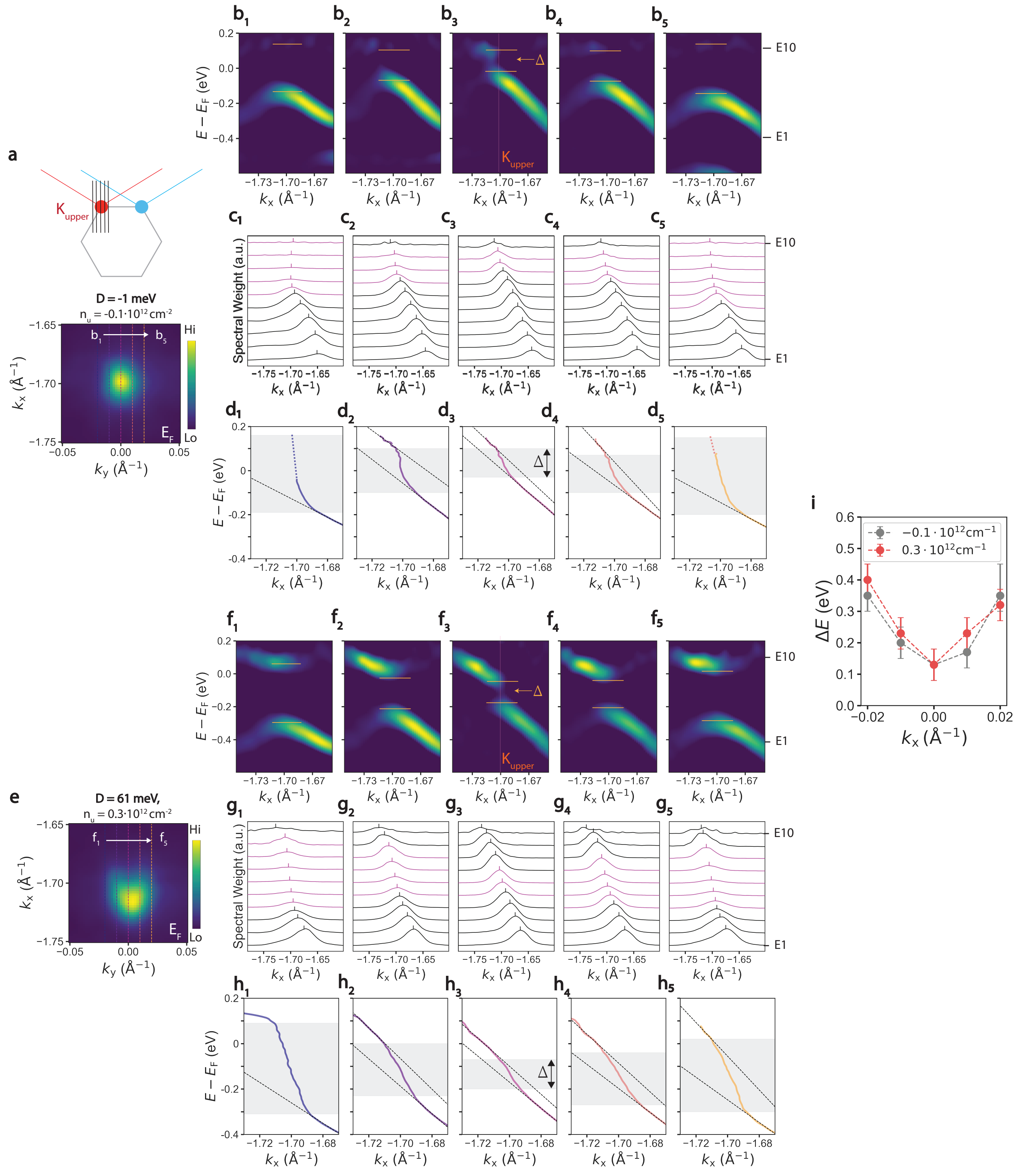

Supplementary Figure 4: Gaps at K_{upper}. (continued on following page)

Supplementary Figure 4: (**a.**) Fermi Surface for 3° twisted graphene at K_{upper} near charge neutrality ($\mathbf{b_1}. - \mathbf{b_5}.$) ARPES second derivative spectra along cuts in momentum space around K_{upper} indicated by vertical dashed lines in **a**. Horizontal orange lines are guides to eye for the top of the valence band and bottom of the conduction band. ($\mathbf{c_1}. - \mathbf{c_5}.$) Corresponding raw MDCs spectra for $\mathbf{b_1} - \mathbf{b_5}$ between energy E1 and E10. Black tick marks indicate quasiparticle peaks with dispersion from the Dirac cone, purple ticks indicate dispersionless peaks. ($\mathbf{d_1}. - \mathbf{d_5}.$) Corresponding dispersions extracted from raw MDCs raw associated with $\mathbf{b_1} - \mathbf{b_5}$. Dashed black lines indicate linear fits to valence and conduction dispersions, grey rectangles indicate regions in which the MDCs are dispersionless. (**e.**) Fermi Surface for 3° twisted graphene at K_{upper} for an electron doping of $1.0{\cdot}10^{12}$ cm^{-2}. ($\mathbf{f_1}. - \mathbf{f_5}.$) ARPES second derivative spectra along cuts in momentum space around K_{upper} indicated by vertical dashed lines in **e**. Horizontal orange lines are guides to eye for the top of the valence band and bottom of the conduction band. ($\mathbf{g_1}. - \mathbf{g_5}.$) Corresponding raw MDCs spectra for $\mathbf{f_1} - \mathbf{f_5}$ between energy E1 and E10. Black tick marks indicate quasiparticle peaks with dispersion from the Dirac cone, purple ticks indicate dispersionless peaks. ($\mathbf{h_1}. - \mathbf{h_5}.$) Corresponding dispersions extracted from raw MDCs raw associated with $\mathbf{f_1} - \mathbf{f_5}$. Dashed black lines indicate linear fits to valence and conduction dispersions, grey rectangles indicate regions in which the MDCs are dispersionless. (**i.**) Summary of the energy separation between top of valence band and bottom of conduction band for each cut in **b** and **f**. Gaps are determined by the minimum band separation at K_{upper}. Error bars are estimated from the experimental energy resolution.

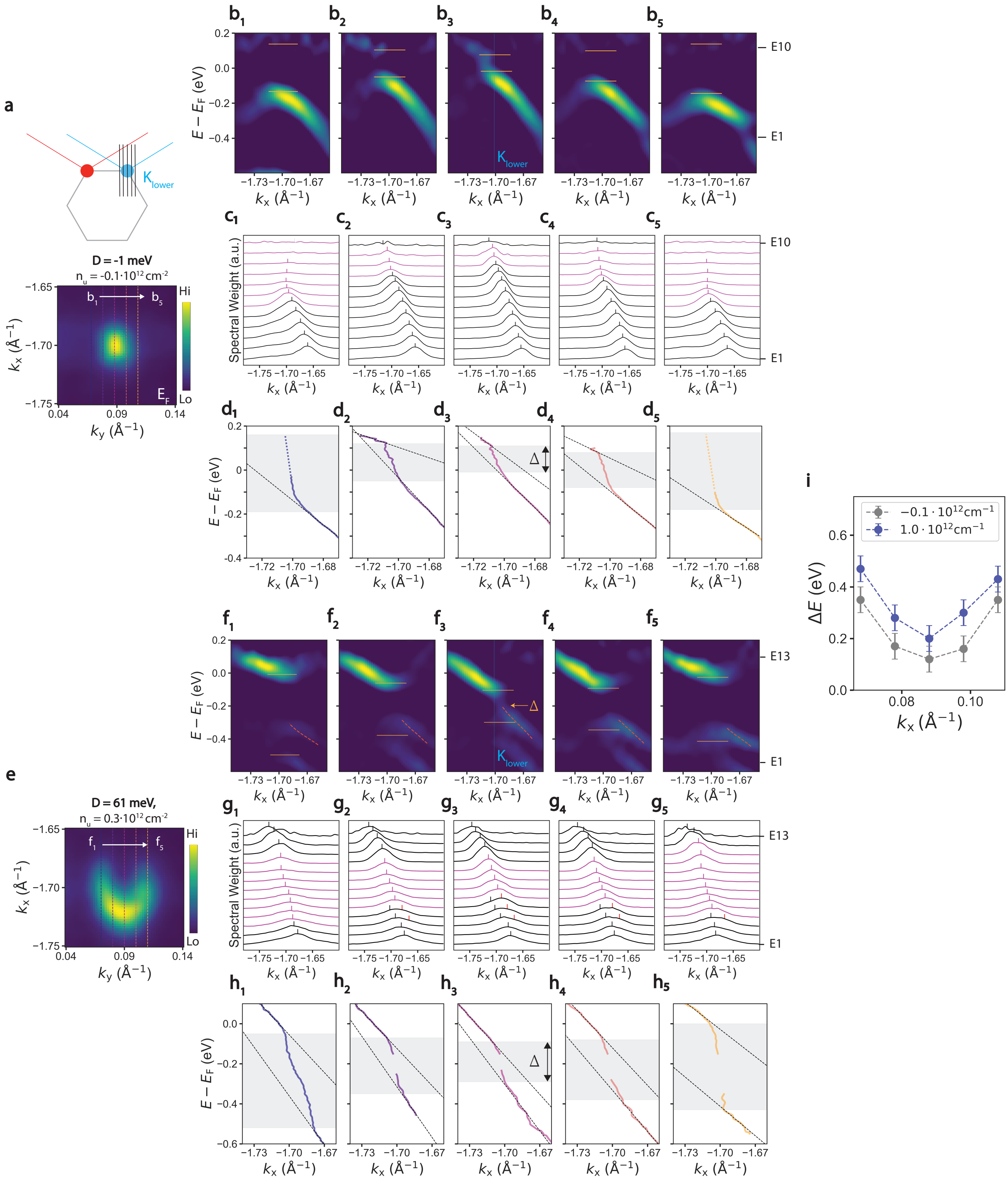

Supplementary Figure 5: Gaps at K_{lower}. (continued on following page)

Supplementary Figure 5: (**a.**) Fermi Surface for 3° twisted graphene at K_{lower} near charge neutrality ($\mathbf{b_1}. - \mathbf{b_5}.$) ARPES second derivative spectra along cuts in momentum space around K_{lower} indicated by vertical dashed lines in **a**. Horizontal orange lines are guides to eye for the top of the valence band and bottom of the conduction band. ($\mathbf{c_1}. - \mathbf{c_5}.$) Corresponding raw MDCs spectra for $\mathbf{b_1} - \mathbf{b_5}$ between energy E1 and E10. Black tick marks indicate quasiparticle peaks with dispersion from the Dirac cone, purple ticks indicate dispersionless peaks. ($\mathbf{d_1}. - \mathbf{d_5}.$) Corresponding dispersions extracted from raw MDCs raw associated with $\mathbf{b_1} - \mathbf{b_5}$. Dashed black lines indicate linear fits to valence and conduction dispersions, grey rectangles indicate regions in which the MDCs are dispersionless. (**e.**) Fermi Surface for 3° twisted graphene at K_{lower} for an electron doping of $1.0 \cdot 10^{12}$ cm^{-2}. ($\mathbf{f_1}. - \mathbf{f_5}.$) ARPES second derivative spectra along cuts in momentum space around K_{upper} indicated by vertical dashed lines in **e**. Horizontal orange lines are guides to eye for the top of the valence band and bottom of the conduction band. Red dashed curves indicate dispersion from Dirac cone replicas of K_{upper} electronic structure. ($\mathbf{g_1}. - \mathbf{g_5}.$) Corresponding raw MDCs spectra for $\mathbf{f_1} - \mathbf{f_5}$ between energy E1 and E10. Black tick marks indicate quasiparticle peaks with dispersion from the Dirac cone, purple ticks indicate dispersionless peaks, red ticks indicate peaks from Dirac cone replicas of K_{upper} electronic structure. ($\mathbf{h_1}. - \mathbf{h_5}.$) Corresponding dispersions extracted from raw MDCs raw associated with $\mathbf{f_1} - \mathbf{f_5}$. Dashed black lines indicate linear fits to valence and conduction dispersions, grey rectangles indicate regions in which the MDCs are dispersionless. (**i.**) Summary of the energy separation between top of valence band and bottom of conduction band for each cut in **b** and **f**. Gaps are determined by the minimum band separation at K_{lower}. Error bars are estimated from the experimental energy resolution.

5. Twist Angle Inhomogeneity Effects on Electronic Structure

We can estimate the twist angle homogeneity of the sample measured in this report using spatially resolved ARPES. Fig 6b,c presents ARPES spectra from two different sample regions (indicated by black and white circles in panel a), separated by over a range of 3 μm, or 3 beam spots. As mentioned in the main text, the twist angle can be measured in ARPES from the momentum separation of the K points of the two graphene layers using the relationship $\Delta K = 2|K| \sin\theta/2$. Panels b and c correspond twist angles of 3° and

2.3°,respectively, corresponding to a moiré wavelength of 6nm and 4nm and a momentum separation $\Delta K \approx 0.09\text{Å}^{-1}$ and $\Delta K \approx 0.07\text{Å}^{-1}$. Clearly, the sample has a spatially inhomogeneous twist angle. Since distinct electronic structure can be seen for the two sample regions, the electronic structure appears to be locally homogeneous over the length scale of the 1 μm beam spot, similar to findings in the literature[40].

Twist angle inhomogeneity often corresponds to doping inhomogeneity, both of which are present in tear-and-stack tw-BLG samples in the literature[10, 40, 41] as well as our sample measured in this report. The careful reader will notice that the valence band Dirac point is above E_{F} for the 3° region, whereas it is not for the 2.3° region, indicating that the doping is different in the two regions of the sample. We investigate this doping inhomogeneity more carefully by examining the size of the Fermi Surface as a function of Gate Voltage (see panels e,f). As mentioned in Supplementary Note 1, the Fermi Surface for each graphene layer is a circle with a radius of the Fermi wavevector k_{F}, which is half the distance between spectral peaks in the MDCs at the Fermi level. The charge neutrality point, determined by the gate voltage at which minimum peak separation in MDCs at E_{F} occurs, is different for the two samples, occurring around 1.5V for the 3° region, and 0.5V for the 2.3° region. This is confirmed by the carrier density as a function of doping in panel g, extracted using Luttinger's Theroeom, i.e. $n_{\mathrm{e}} = k_{\mathrm{F}}^2/\pi$, which has a minimum at 1.5V for the 3° region, and 0.5V for the 2.3° region. Such doping inhomogeneity present in our sample may provide an additional explanation for why macroscopic measurements such as transport do not find very large gaps at the neutrality point[42, 43]: since different regions have different dopings, one sample region that is in the gap and insulating will be adjacent to another sample region that has carriers populated and thus is conducting.

To our knowledge the local gap size is not strongly affected by the twist angle within this particular twist angle range measured in the experiment. Fig 6h and i present equilibrium ARPES second derivative spectra along $\Gamma - \mathrm{K}_{\mathrm{upper}}$ for the 3° and 2.3° regions, respectively. Both twist angle regions exhibit $\approx$ 100 meV separation between conduction and valence bands, which is confirmed by the region in energy where there is deviation from linearity in the dispersions (right sides of panels h and i). Indeed, the strain that produces the twist angle inhomogeneity on our sample clearly does not have such a strong affect on the gap size at the neutralty point in our experimental conditions, as suggested by early theoretical works on tw-BLG [44]. This is clearly not the case for the magic angle twist regime at

low temperature, where there is a competition between Kekule spiral order and K-IVC states[45, 46].

6. Spatial Inhomogeneity Effects on Carrier Density Measurements

The spatial inhomogeneity ubiquitous in materials often precludes bulk probes such as transport from producing accurate readings of sample carrier concentration. Typically, this shows up when the surface carrier density is different from that of the bulk, e.g. from surface dosing [47, 48], or the presence of surface and interface states[49, 50] present on bulk conductive materials. A local and surface sensitive probe such as nanoARPES is therefore much better suited to access the carrier density because, as mentioned in the previous note, it can locally measure Luttinger volume of the Fermi surface[47, 49, 50].

The spatial inhomogeneity of our sample comes into account when estimating the carrier density using a simple capacitance model, i.e. $n = C(n)V_g$ where the capacitance $C(n)$ is determined by $\frac{1}{C} = \frac{1}{C_g} + \frac{1}{C_q}$, and C_g and C_q are the geometric and quantum contributions to the capacitance, respectively [51]. Let's focus first on the geometric capacitance, which dominates away from charge neutrality when our sample is more or less metallic: the hBN contribution is given by $C_{g,hBN} = \epsilon_{hBN}\epsilon_0/d_{hBN} \approx 0.19\frac{\mu\mathrm{F}}{\mathrm{cm}^2}$ for an hBN thickness of 15nm and dielectric strength $\epsilon_{hBN} = 3.4$. Interestingly, this value vastly overestimates the carrier concentration obtained via ARPES (see Fig 7d away from neutrality).

We attribute this discrepancy to the presence of an air gap between the sample and the graphite back gate, perhaps due to the proximity of measurement locations to a wrinkle on hBN (pink dashed line in Fig 7a). Indeed, a recent study of the hBN dielectric strength found that such air gaps develop in about a third of devices, substantially decreasing the overall effective dielectric strength of the capacitor[52]. The air gap behaves as a capacitor in series, i.e. $\frac{1}{C_g} = \frac{d}{\epsilon_r} = \frac{1}{C_{g,hBN}} + \frac{1}{C_{g,air}}$. By plugging in dielectric strengths of 3.4[52] for hBN and 1 for air, and a thickness of $d_{\mathrm{hBN}} = 15$nm (see panel b), we estimate that an 8nm air gap is present between the hBN and our sample, producing an overall effective dielectric strength of $\epsilon_r = 2$, resulting in doping behavior presented in Fig 7c. Overall, the spatial inhomogeneity present in our twisted graphene/hBN heterostructure in the form of an air gap results in an inaccurate estimate of the carrier density, which is resolved using nanoARPES.

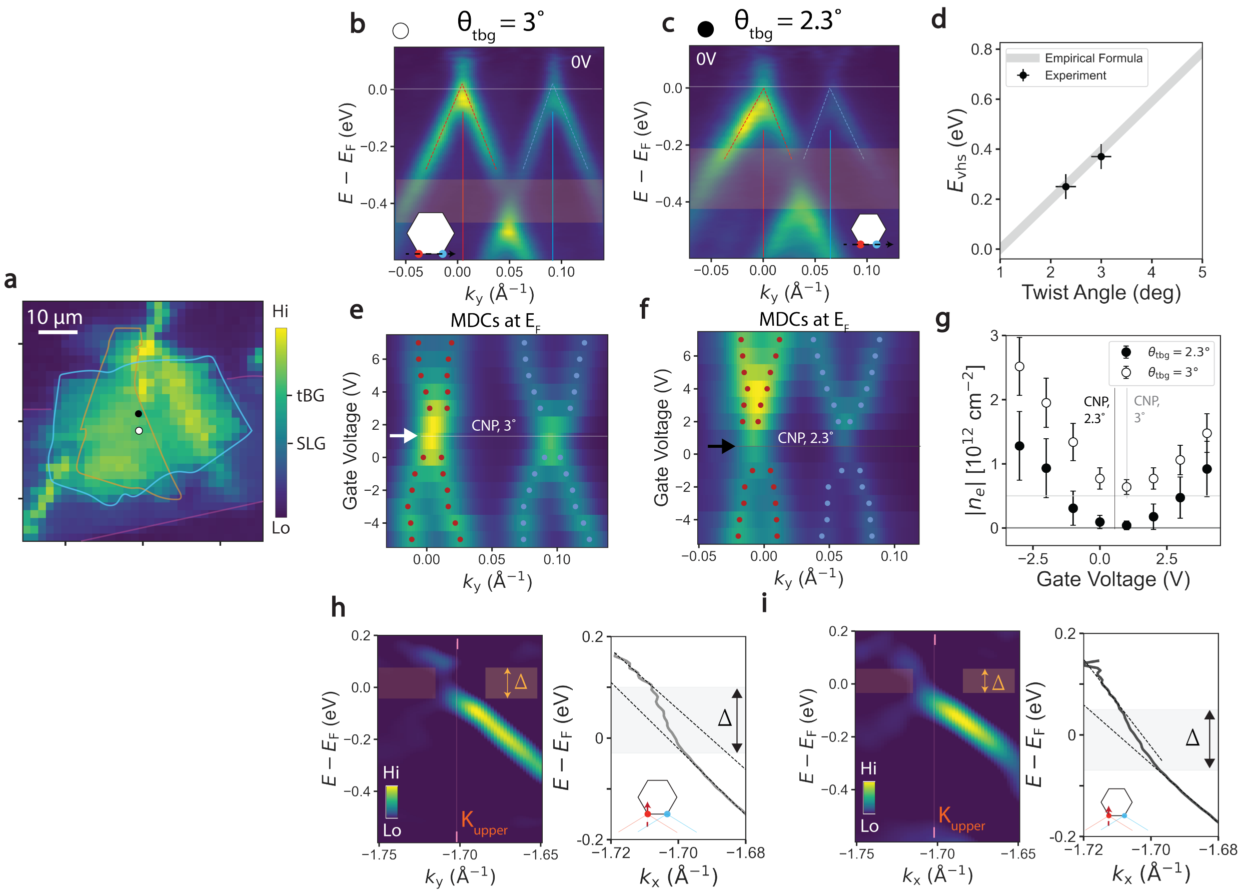

Supplementary Figure 6: Twist Angle Inhomogeneity Effects on Electronic Structure. (**a.**) scanning photoemission microscopy (SPEM) image integrated over states at E_F, from Fig 1 in main text. (**b., c.**) Corresponding equilibrium ARPES spectra along $K_\mathrm{upper} - K_\mathrm{lower}$ direction at locations indicated by white (**b-**) and black (**c-**) circles in (**a**). Red (blue) dashed lines indicate linear fits to quasiparticle peak positions extracted from Lorentzian fits. Shaded orange region indicates hybridization minigap formed at the intersection of the two Dirac cones. (**d**) van Hove singularity binding energy as a function of twist angle, extracted from ARPES data (black) and compared with an empirical formula for intersecting Dirac cones (grey). (**e., f.**) MDCs at E_F as a function of gate voltage for the 3° (**e-**) and 2.3° (**f-**) samples. Red (blue) circles indicate quasiparticle peak positions for the upper (lower) K point spectra extracted from Loretnzian peak fits. White (Black) lines indicate estimates for charge neutrality points for the 3° (2.3°) sample. (continued on following page).

Supplementary Figure 6: (**g.**) Carrier density n_{e} as a function of gate voltage for the 3° (white, offset by $0.5{\cdot}10^{12}\,\mathrm{cm}^{-2}$) and 2.3° (black) samples, calculated using Luttinger's theorem: $n_{\mathrm{e}} = {k_{\mathrm{F}}}^2/\pi$. Error bars represent errors propagated from estimates of k_{F}. Vertical black (grey) lines indicate estimate of charge neutrality point for the 3° (2.3°) sample. (**h.**) 3° twisted graphene second derivative spectra (left) and corresponding quasiparticle dispersion (right) along $\Gamma - \mathrm{K}_{\mathrm{upper}}$. Gaps in the dispersion are indicated by orange (left) and grey (right) shaded regions, which are bordered by kinks in the MDCs dispersion. Dashed lines indicate linear fits to the conduction and valence dispersions that highlight these borders. (**i.**) same analysis as (**h**) for the 2.3° twisted graphene region.

Finally, we comment briefly on the quantum capacitance contributions, which occur at low carrier density. Quantum capacitance is general given by the density of states i.e. $C_q = e^2 D(E)$, and in a massless Dirac fermion model of twisted bilayer graphene this is given by $C_q = \frac{e^2 2\sqrt{2}}{\sqrt{\pi}\hbar v_F}\sqrt{|n| + n_d}$ where $v_F = 1.2 \cdot 10^6$m/s is the Fermi velocity, and $n_d = 1.0 \cdot 10^1 0\mathrm{cm}^{-2}$ is the excess carrier density present due to disorder[53]. This quantum capacitance model of massless Dirac Fermions (pink dashed line in panel d) overestimates the measured capacitance of our sample near the neutrality point, suggesting the presence of a gap in the band structure, further supporting the evidence provided by the ARPES spectra in the main manuscript.

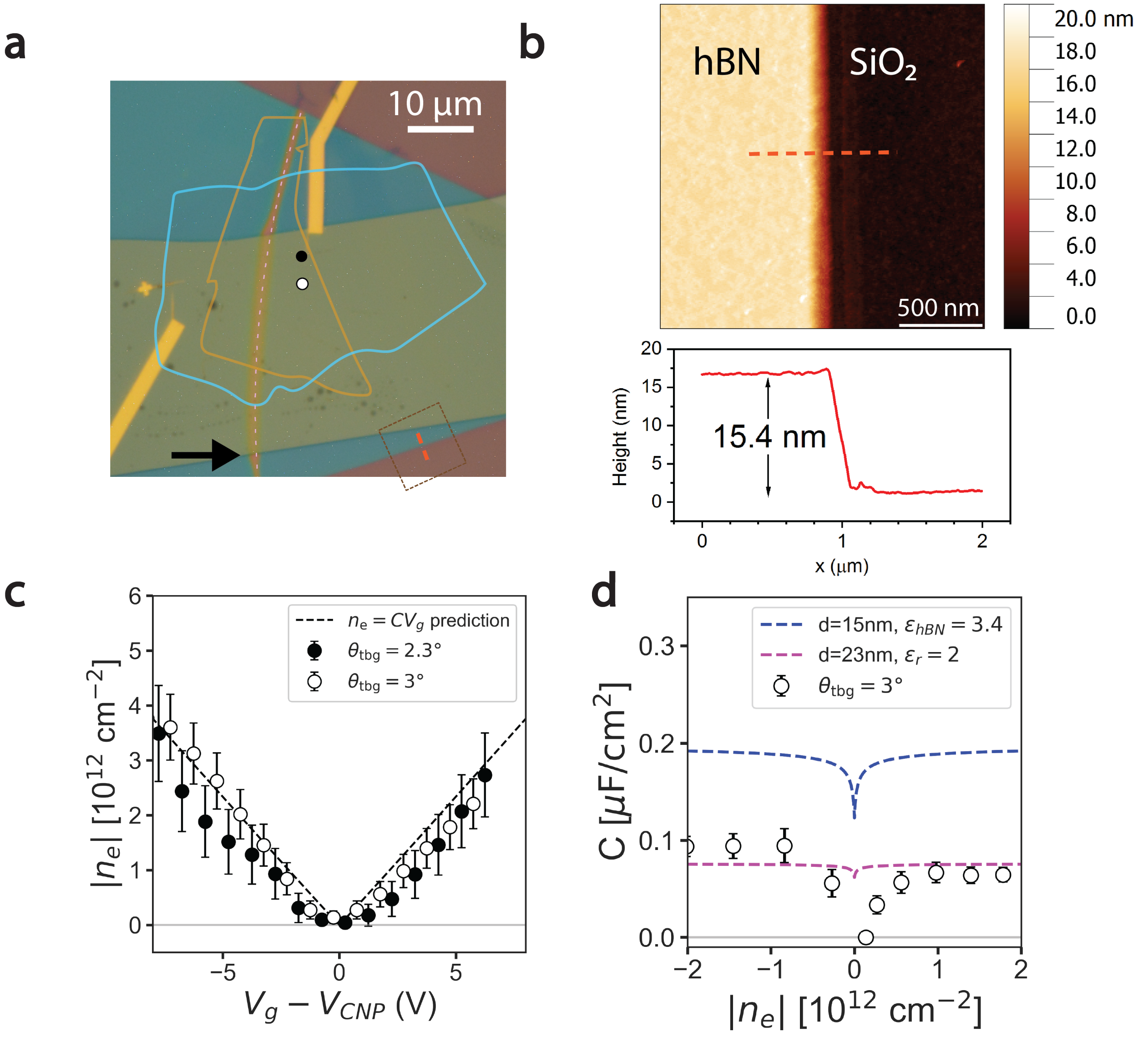

Supplementary Figure 7: Spatial Inhomogeneity Effects on Carrier Density Measurements. (**a.**) Optical micrograph of twisted bilayer graphene/hBN sample. Regions of graphene upper layer (orange), lower layer (blue) are outlined in with false color. Pink dashed curve indicates wrinkle in hBN substrate (**b.**) AFM micrograph of hBN substrate within region outlined by brown square in **a.** (top) and corresponding line cute (bottom), indicating hBN thickness. (**c.**) Carrier density n_e as a function of gate voltage for both sample regions, as compared to a quantum capacitance model described in the text. Both sets of data are offset by the charge neutrality point voltage obtained from Fig 6 (**d.**) Capacitance as a function of carrier density for the 3° sample (white circles). Blue and magenta dashed curves indicate quantum capacitance models of tw-BLG with a pure hBN dielectric, and additional 8nm air gap, respectively.

7. Discussion of the Origin of Doping- and Layer- Dependent Band Velocity and Band Gap Enhancements

The data reported in the main manuscript provide evidence for a method of tuning the band velocities and band gaps in twisted bilayer graphene in operando. As we will argue below, we believe that these effects can be best explained by a combination of the substrate interaction and the spatially inhomogeneous Hartree-Fock interaction, which is controlled within different layers using a displacement field.

First we address the K point dispersions, which can be modified by several different mechanisms, including single particle effects from band displacement and strain, as well as many-body effects such as electron-electron interactions.

On a single particle level, band displacement in twisted bilayer graphene brings the Dirac point of one layer closer in energy to the valence van-Hove-singularity E_{vHs}, and the opposite for the other layer [7, 54], while keeping the overall bandwidth constant. At large displacement ($D \approx E_{vHs}$), the valence band velocity at the Dirac point which is energetically closer to the van Hove singularity decreases, and the band velocity at the Dirac point which is farther from the van Hove singularity increases [54]. However, this behavior is qualitatively inconsistent with our results, as the band velocity at both Dirac points is modified in the same direction upon changing the doping.

Both uniaxial and heterostrain can also change the bandwidth in tw-BLG[43, 44]. Uniaxial strain brings the Dirac points of different layers in closer proximity in momentum space[55], producing an effect similar to that produced by decreasing the overall twist angle [56, 57]. Heterostrain, on the other hand adjusts the interlayer hopping such that the first magic angle condition for flat bands occurs at larger twist angle, and notably resulting in a shift of the Dirac cones from the mini Brillouin zone corners[44]. However, typical strain present in twisted graphene samples is $\approx 1\%$[58], which is an order of magnitude smaller than the 16% strain required to observe the band structure modifications of $\approx 40\%$ in our experiment[55]. Additionally, the Dirac cone momentum separation does not change with application of a gate voltage in our experiment, suggesting that any strain present in our sample is not gate-tunable, and therefore cannot explain the doping behavior of the band velocity.

As mentioned earlier, electron-electron interactions are known causes of significant band

velocity modification both in single layer[9, 13, 14, 59–61] and twisted bilayer [10, 16–18, 62] graphene. In the case of single layer graphene, the band velocity enhancement is strongest at the the charge neutrality point, and weaker for electron and hole-dopings[9, 59, 61], which is inconsistent with our data. On the other hand, the spatially inhomogeneous Hartree Fock interaction in twisted blayer graphene qualitatively matches the doping dependence seen in our data at the K points: i.e. a steady valence band velocity decrease with hole-doping[10, 11]. We therefore attribute the doping behavior of the K point dispersions to such Hartree-Fock interactions.

We now address the gap present in our data. Several mechanisms can produce a band separation in graphene-based systems, including many body interactions, interlayer potentials, and inversion symmetry breaking. The electron-plasmon interaction, for example, has been demonstrated to generate a plasmaron satellite in highly doped graphene systems[63–66], which can take the appearance of a gap. However, the doping dependence of the satellite separation for a plasmaron is linear in E_{F}, and therefore it cannot explain the presence of the gap at the neutrality point at zero doping. Additionally, the plasmaron has been predicted[67] and demonstrated[66, 68] to compete with gaps, as in the case of graphene aligned to hBN[66].

Interlayer potentials may also produce band gaps in twisted bilayer graphene. At particular commensurate twist angles, the K points from each layer become equivalent points in the mini Brillouin zone , resulting in an umklapp scattering gap whose magnitude scales positively with the twist angle [69–74]. However, our sample has a twist angle of 3°, rendering it among the twist angles where $\mathrm{K}_{\mathrm{upper}}$ and $\mathrm{K}_{\mathrm{lower}}$ are not connected by a reciprocal lattice vector and such umklapp scattering is not predicted [71, 72].

Inversion symmetry breaking, is well known to produce band gaps in graphene [8, 75, 76] and commonly occurs in the presence of a substrate with a different lattice constant[8, 76–79], even when the substrate is crystallographically misaligned [76, 78, 79] as in the case of our sample. Though a gap from a misaligned hBN substrate may be small[78, 80], interactions may enhance them significantly[80, 81]. For example, electron-electron interactions enhance band gaps in graphene with a magnitude that scales with the interaction strength[80–82], which in tBG scales linearly with doping [16–18, 62]. Upon generating a charge imbalance between the two graphene layers, e.g. via a displacement field, this mechanism enables layer-dependent gap enhancements such as those seen in our experiment.

These observations make the substrate and Hartree-Fock interactions the most likely candidates for generating and enhancing the band gap in 3° twisted bilayer graphene. Indeed these interactions can qualitatively explain both the presence of the gap at charge neutrality as well as the layer-dependent gap enhancement upon electron doping the sample.

III. SUPPLEMENTARY REFERENCES

[1] M. Iqbal Bakti Utama, Roland J. Koch, Kyunghoon Lee, Nicolas Leconte, Hongyuan Li, Sihan Zhao, Lili Jiang, Jiayi Zhu, Kenji Watanabe, Takashi Taniguchi, Paul D. Ashby, Alexander Weber-Bargioni, Alex Zettl, Chris Jozwiak, Jeil Jung, Eli Rotenberg, Aaron Bostwick, and Feng Wang, "Visualization of the flat electronic band in twisted bilayer graphene near the magic angle twist," Nature Physics **17**, 184–188 (2021).

[2] Roland J. Koch, Chris Jozwiak, Aaron Bostwick, Benjamin Stripe, Mark Cordier, Zahid Hussain, Wenbing Yun, and Eli Rotenberg, "Nano focusing of soft x-rays by a new capillary mirror optic," Synchrotron Radiation News **31**, 50–52 (2018).

[3] Conrad Stansbury and Alessandra Lanzara, "PyARPES: An analysis framework for multimodal angle-resolved photoemission spectroscopies," SoftwareX **11**, 100472 (2020).

[4] J. D. Rameau, H. B. Yang, and P. D. Johnson, "Application of the Lucy-Richardson deconvolution procedure to high resolution photoemission spectra," Journal of Electron Spectroscopy and Related Phenomena **181**, 35–43 (2010).

[5] Yuan Cao, Valla Fatemi, Shiang Fang, Kenji Watanabe, Takashi Taniguchi, Efthimios Kaxiras, and Pablo Jarillo-Herrero, "Unconventional superconductivity in magic-angle graphene superlattices," Nature **556**, 43–50 (2018).

[6] Alfred J.H. Jones, Ryan Muzzio, Paulina Majchrzak, Sahar Pakdel, Davide Curcio, Klara Volckaert, Deepnarayan Biswas, Jacob Gobbo, Simranjeet Singh, Jeremy T. Robinson, Kenji Watanabe, Takashi Taniguchi, Timur K. Kim, Cephise Cacho, Nicola Lanata, Jill A. Miwa, Philip Hofmann, Jyoti Katoch, and Søren Ulstrup, "Observation of Electrically Tunable van Hove Singularities in Twisted Bilayer Graphene from NanoARPES," Advanced Materials **2001656**, 1–7 (2020).

[7] Chao Hui Yeh, Yung Chang Lin, Yu Chen Chen, Chun Chieh Lu, Zheng Liu, Kazu Suenaga, and Po Wen Chiu, "Gating electron-hole asymmetry in twisted bilayer graphene," ACS Nano **8**, 6962–6969 (2014).

[8] Eryin Wang, Xiaobo Lu, Shijie Ding, Wei Yao, Mingzhe Yan, Guoliang Wan, Ke Deng, Shuopei Wang, Guorui Chen, Liguo Ma, Jeil Jung, Alexei V. Fedorov, Yuanbo Zhang, Guangyu Zhang, and Shuyun Zhou, "Gaps induced by inversion symmetry breaking and second-generation Dirac cones in graphene/hexagonal boron nitride," Nature Physics **12**, 1111–1115 (2016).

[9] Nicholas Dale, Ryo Mori, M. Iqbal Bakti Utama, Jonathan D. Denlinger, Conrad Stansbury, Claudia G. Fatuzzo, Sihan Zhao, Kyunghoon Lee, Takashi Taniguchi, Kenji Watanabe, Chris Jozwiak, Aaron Bostwick, Eli Rotenberg, Roland J. Koch, Feng Wang, and Alessandra Lanzara, "Correlation-driven electron-hole asymmetry in graphene field effect devices," npj Quantum Materials **7**, 1–7 (2022).

[10] Youngjoon Choi, Hyunjin Kim, Cyprian Lewandowski, Yang Peng, Alex Thomson, Robert Polski, Yiran Zhang, Kenji Watanabe, Takashi Taniguchi, Jason Alicea, and Stevan Nadj-Perge, "Interaction-driven band flattening and correlated phases in twisted bilayer graphene," Nature Physics **17**, 1375–1381 (2021).

[11] Cyprian Lewandowski, Stevan Nadj-Perge, and Debanjan Chowdhury, "Does filling-dependent band renormalization aid pairing in twisted bilayer graphene?" npj Quantum Materials **6**, 82 (2021).

[12] Zachary A.H. Goodwin, Valerio Vitale, Xia Liang, Arash A. Mostofi, and Johannes Lischner, "Hartree theory calculations of quasiparticle properties in twisted bilayer grapheme," Electronic Structure **2**, 034001 (2020).

[13] J. González, F. Guinea, and M. A.H. Vozmediano, "Non-Fermi liquid behavior of electrons in the half-filled honeycomb lattice (A renormalization group approach)," Nuclear Physics, Section B **424**, 595–618 (1994).

[14] D. A. Siegel, C.-H. Park, C. Hwang, J. Deslippe, A. V. Fedorov, S. G. Louie, and A. Lanzara, "Many-body interactions in quasi-freestanding graphene," Proceedings of the National Academy of Sciences **108**, 11365–11369 (2011).

[15] Choongyu Hwang, David A. Siegel, Sung Kwan Mo, William Regan, Ariel Ismach, Yuegang Zhang, Alex Zettl, and Alessandra Lanzara, "Fermi velocity engineering in graphene by substrate modification," Scientific Reports **2**, 2–5 (2012).

[16] Francisco Guinea and Niels R. Walet, "Electrostatic effects, band distortions, and superconductivity in twisted graphene bilayers," Proceedings of the National Academy of Sciences of the United States of America **115**, 13174–13179 (2018).

[17] Tommaso Cea, Niels R. Walet, and Francisco Guinea, "Electronic band structure and pinning of Fermi energy to Van Hove singularities in twisted bilayer graphene: A self-consistent approach," Physical Review B **100**, 205113 (2019).

[18] Tommaso Cea and Francisco Guinea, "Band structure and insulating states driven by Coulomb interaction in twisted bilayer graphene," Physical Review B **102**, 045107 (2020).

[19] Jihang Zhu, Jingtian Shi, and Allan H. MacDonald, "Theory of angle-resolved photoemission spectroscopy in graphene-based moiré superlattices," Physical Review B **103**, 235146 (2021).

[20] Nicolas Leconte, Srivani Javvaji, Jiaqi An, Appalakondaiah Samudrala, and Jeil Jung, "Relaxation effects in twisted bilayer graphene: A multiscale approach," Physical Review B **106**, 115410 (2022).

[21] Mingjian Wen, Yaser Afshar, Ryan S. Elliott, and Ellad B. Tadmor, "KLIFF: A framework to develop physics-based and machine learning interatomic potentials," Computer Physics Communications **272**, 108218 (2022).

[22] Mingjian Wen, Stephen Carr, Shiang Fang, Efthimios Kaxiras, and Ellad B. Tadmor, "Dihedral-angle-corrected registry-dependent interlayer potential for multilayer graphene structures," Physical Review B **98**, 235404 (2018).

[23] Nicolas Leconte, Jeil Jung, Sébastien Lebègue, and Tim Gould, "Moiré-pattern interlayer potentials in van der waals materials in the random-phase approximation," Phys. Rev. B **96**, 195431 (2017).

[24] Steve Plimpton, "Fast Parallel Algorithms for Short-Range Molecular Dynamics," Journal of Computational Physics **117**, 1–19 (1995).

[25] Donald W Brenner, Olga A Shenderova, Judith A Harrison, Steven J Stuart, Boris Ni, and Susan B Sinnott, "A second-generation reactive empirical bond order (REBO) potential energy expression for hydrocarbons," Journal of Physics: Condensed Matter **14**, 783 (2002).

[26] Jeil Jung and Allan H. Macdonald, "Tight-binding model for graphene π-bands from maximally localized Wannier functions," Physical Review B - Condensed Matter and Materials Physics **87**, 1–10 (2013).

[27] Nicolas Leconte, Srivani Javvaji, Jiaqi An, Appalakondaiah Samudrala, and Jeil Jung, "Re-

laxation effects in twisted bilayer graphene: A multiscale approach," Phys. Rev. B **106**, 115410 (2022).

[28] G. Trambly de Laissardiére, D. Mayou, and L. Magaud, "Numerical studies of confined states in rotated bilayers of graphene," Physical Review B **86**, 125413 (2012).

[29] Wei Ku, Tom Berlijn, and Chi-Cheng Lee, "Unfolding first-principles band structures," Phys. Rev. Lett. **104**, 216401 (2010).

[30] Hirofumi Nishi, Yu Ichiro Matsushita, and Atsushi Oshiyama, "Band-unfolding approach to moiré-induced band-gap opening and Fermi level velocity reduction in twisted bilayer graphene," Physical Review B **95**, 1–8 (2017).

[31] Yu-ichiro Matsushita, Hirofumi Nishi, Jun-ichi Iwata, Taichi Kosugi, and Atsushi Oshiyama, "Unfolding energy spectra of double-periodicity two-dimensional systems: Twisted bilayer graphene and MoS2 on graphene," Physical Review Materials **2**, 010801 (2018).

[32] B. Amorim, "General theoretical description of angle-resolved photoemission spectroscopy of van der waals structures," Phys. Rev. B **97**, 165414 (2018).

[33] M. Mucha-Kruczyński, J. R. Wallbank, and V. I. Fal'ko, "Moiré miniband features in the angle-resolved photoemission spectra of graphene/hBN heterostructures," Phys. Rev. B **93**, 085409 (2016).

[34] Jihang Zhu, Jingtian Shi, and Allan H. MacDonald, "Theory of angle-resolved photoemission spectroscopy in graphene-based moiré superlattices," Phys. Rev. B **103**, 235146 (2021).

[35] B. Amorim and Eduardo V. Castro, "Electronic spectral properties of incommensurate twisted trilayer graphene," arXiV:1807.11909 Cond-Matter , 1–8 (2018).

[36] Choongyu Hwang, Cheol-Hwan Park, David A. Siegel, Alexei V. Fedorov, Steven G. Louie, and Alessandra Lanzara, "Direct measurement of quantum phases in graphene via photoemission spectroscopy," Phys. Rev. B **84**, 125422 (2011).

[37] Sohrab Ismail-Beigi, Eric K. Chang, and Steven G. Louie, "Coupling of nonlocal potentials to electromagnetic fields," Phys. Rev. Lett. **87**, 087402 (2001).

[38] S. Y. Zhou, D. A. Siegel, A. V. Fedorov, and A. Lanzara, "Departure from the conical dispersion in epitaxial graphene," Physica E: Low-Dimensional Systems and Nanostructures **40**, 2642–2647 (2008).

[39] Paul V. Nguyen, Natalie C. Teutsch, Nathan P. Wilson, Joshua Kahn, Xue Xia, Abigail J. Graham, Viktor Kandyba, Alessio Giampietri, Alexei Barinov, Gabriel C. Constantinescu,

Nelson Yeung, Nicholas D. M. Hine, Xiaodong Xu, David H. Cobden, and Neil R. Wilson, "Visualizing electrostatic gating effects in two-dimensional heterostructures," Nature **572**, 220–223 (2019).

[40] Andrew T. Pierce, Yonglong Xie, Jeong Min Park, Eslam Khalaf, Seung Hwan Lee, Yuan Cao, Daniel E. Parker, Patrick R. Forrester, Shaowen Chen, Kenji Watanabe, Takashi Taniguchi, Ashvin Vishwanath, Pablo Jarillo-Herrero, and Amir Yacoby, "Unconventional sequence of correlated Chern insulators in magic-angle twisted bilayer graphene," Nature Physics **17**, 1210–1215 (2021).

[41] Youngjoon Choi, Hyunjin Kim, Yang Peng, Alex Thomson, Cyprian Lewandowski, Robert Polski, Yiran Zhang, Harpreet Singh Arora, Kenji Watanabe, Takashi Taniguchi, Jason Alicea, and Stevan Nadj-Perge, "Correlation-driven topological phases in magic-angle twisted bilayer graphene," Nature **589**, 536–541 (2021).

[42] Y. Cao, J. Y. Luo, V. Fatemi, S. Fang, J. D. Sanchez-Yamagishi, K. Watanabe, T. Taniguchi, E. Kaxiras, and P. Jarillo-Herrero, "Superlattice-Induced Insulating States and Valley-Protected Orbits in Twisted Bilayer Graphene," Physical Review Letters **117**, 1–5 (2016).

[43] Le Zhang, Ying Wang, Rendong Hu, Puhua Wan, Oleksandr Zheliuk, Minpeng Liang, Xiaoli Peng, Yu Jia Zeng, and Jianting Ye, "Correlated States in Strained Twisted Bilayer Graphenes Away from the Magic Angle," Nano Letters **22**, 3204–3211 (2022).

[44] Zhen Bi, Noah F.Q. Yuan, and Liang Fu, "Designing flat bands by strain," Physical Review B **100**, 1–9 (2019).

[45] Yonglong Xie, Andrew T. Pierce, Jeong Min Park, Daniel E. Parker, Eslam Khalaf, Patrick Ledwith, Yuan Cao, Seung Hwan Lee, Shaowen Chen, Patrick R. Forrester, Kenji Watanabe, Takashi Taniguchi, Ashvin Vishwanath, Pablo Jarillo-Herrero, and Amir Yacoby, "Fractional Chern insulators in magic-angle twisted bilayer graphene," Nature **600**, 439–443 (2021).

[46] Kevin P. Nuckolls, Ryan L. Lee, Myungchul Oh, Dillon Wong, Tomohiro Soejima, Jung Pyo Hong, Dumitru Călugăru, Jonah Herzog-Arbeitman, B Andrei Bernevig, Kenji Watanabe, Takashi Taniguchi, Nicolas Regnault, Michael P. Zaletel, and Ali Yazdani, "Quantum textures of the many-body wavefunctions in magic-angle graphene," arXiV:2303.00024 Cond-Matter , 1–21 (2023).

[47] C. H.P. Wen, H. C. Xu, C. Chen, Z. C. Huang, X. Lou, Y. J. Pu, Q. Song, B. P. Xie, Mahmoud Abdel-Hafiez, D. A. Chareev, A. N. Vasiliev, R. Peng, and D. L. Feng, "Anomalous

correlation effects and unique phase diagram of electron-doped FeSe revealed by photoemission spectroscopy," Nature Communications **7**, 1–7 (2016).

[48] L. J. Li, E. C.T. O'Farrell, K. P. Loh, G. Eda, B. Özyilmaz, and A. H. Castro Neto, "Controlling many-body states by the electric-field effect in a two-dimensional material," Nature **529**, 185–189 (2016).

[49] D. Hsieh, Y. Xia, D. Qian, L. Wray, J. H. Dil, F. Meier, J. Osterwalder, L. Patthey, J. G. Checkelsky, N. P. Ong, A. V. Fedorov, H. Lin, A. Bansil, D. Grauer, Y. S. Hor, R. J. Cava, and M. Z. Hasan, "A tunable topological insulator in the spin helical Dirac transport regime," Nature **460**, 1101–1105 (2009).

[50] Siobhan McKeown Walker, Margherita Boselli, Emanuel A. Martínez, Stefano Gariglio, Flavio Y. Bruno, and Felix Baumberger, "A Laser-ARPES View of the 2D Electron Systems at LaAlO3/SrTiO3 and Al/SrTiO3 Interfaces," Advanced Electronic Materials **8**, 1–8 (2022).

[51] G. L. Yu, R. Jalil, Branson Belle, Alexander S. Mayorov, Peter Blake, Frederick Schedin, Sergey V. Morozov, Leonid A. Ponomarenko, F. Chiappini, S. Wiedmann, Uli Zeitler, Mikhail I. Katsnelson, A. K. Geim, Kostya S. Novoselov, and Daniel C. Elias, "Interaction phenomena in graphene seen through quantum capacitance," Proceedings of the National Academy of Sciences of the United States of America **110**, 3282–3286 (2013).

[52] A. Pierret, D. Mele, H. Graef, J. Palomo, T. Taniguchi, K. Watanabe, Y. Li, B. Toury, C. Journet, P. Steyer, V. Garnier, A. Loiseau, J. M. Berroir, E. Bocquillon, G. Fève, C. Voisin, E. Baudin, M. Rosticher, and B. Plaçais, "Dielectric permittivity, conductivity and breakdown field of hexagonal boron nitride," Materials Research Express **9**, 065901 (2022).

[53] Yuan Cao, Valla Fatemi, Ahmet Demir, Shiang Fang, Spencer L Tomarken, Jason Y Luo, Javier D. Sanchez-Yamagishi, Kenji Watanabe, Takashi Taniguchi, Efthimios Kaxiras, Ray C Ashoori, and Pablo Jarillo-Herrero, "Correlated insulator behaviour at half-filling in magic-angle graphene superlattices," Nature **556**, 80–84 (2018).

[54] Guy Trambly De Laissardière, Omid Faizy Namarvar, Didier Mayou, and Laurence Magaud, "Electronic properties of asymmetrically doped twisted graphene bilayers," Physical Review B **93**, 1–12 (2016).

[55] Wei Yan, Wen Yu He, Zhao Dong Chu, Mengxi Liu, Lan Meng, Rui Fen Dou, Yanfeng Zhang, Zhongfan Liu, Jia Cai Nie, and Lin He, "Strain and curvature induced evolution of electronic

band structures in twisted graphene bilayer," Nature Communications **4**, 1–7 (2013).

[56] J. M.B. Lopes Dos Santos, N. M.R. Peres, and A. H. Castro Neto, "Graphene bilayer with a twist: Electronic structure," Physical Review Letters **99**, 19–22 (2007).

[57] R. Bistritzer and A. H. MacDonald, "Moire bands in twisted double-layer graphene," Proceedings of the National Academy of Sciences **108**, 12233–12237 (2011).

[58] Alexander Kerelsky, Leo J. McGilly, Dante M. Kennes, Lede Xian, Matthew Yankowitz, Shaowen Chen, K. Watanabe, T. Taniguchi, James Hone, Cory Dean, Angel Rubio, and Abhay N. Pasupathy, "Maximized electron interactions at the magic angle in twisted bilayer graphene," Nature **572**, 95–100 (2019).

[59] D. C. Elias, R. V. Gorbachev, A. S. Mayorov, S. V. Morozov, A. A. Zhukov, P. Blake, L. A. Ponomarenko, I. V. Grigorieva, K. S. Novoselov, F. Guinea, and A. K. Geim, "Dirac cones reshaped by interaction effects in suspended graphene," Nature Physics **7**, 701–704 (2011).

[60] S. Das Sarma, E. H. Hwang, and Wang-Kong Tse, "Many-body interaction effects in doped and undoped graphene: Fermi liquid versus non-Fermi liquid," Physical Review B **75**, 121406 (2007).

[61] David A. Siegel, William Regan, Alexei V. Fedorov, A. Zettl, and Alessandra Lanzara, "Charge-Carrier Screening in Single-Layer Graphene," Physical Review Letters **110**, 146802 (2013).

[62] Pietro Novelli, Iacopo Torre, Frank H. L. Koppens, Fabio Taddei, and Marco Polini, "Optical and plasmonic properties of twisted bilayer graphene: Impact of interlayer tunneling asymmetry and ground-state charge inhomogeneity," Physical Review B **102**, 125403 (2020).

[63] Marco Polini, Reza Asgari, Giovanni Borghi, Yafis Barlas, T. Pereg-Barnea, and A. H. MacDonald, "Plasmons and the spectral function of graphene," Physical Review B - Condensed Matter and Materials Physics **77**, 3–6 (2008).

[64] Aaron Bostwick, Florian Speck, Thomas Seyller, Karsten Horn, Marco Polini, Reza Asgari, Allan H MacDonald, and Eli Rotenberg, "Observation of Plasmarons in Quasi-Freestanding Doped Graphene," Science **328**, 999–1002 (2010).

[65] Andrew L. Walter, Aaron Bostwick, Ki Joon Jeon, Florian Speck, Markus Ostler, Thomas Seyller, Luca Moreschini, Young Jun Chang, Marco Polini, Reza Asgari, Allan H. MacDonald, Karsten Horn, and Eli Rotenberg, "Effective screening and the plasmaron bands in graphene," Physical Review B - Condensed Matter and Materials Physics **84**, 1–8 (2011).

[66] Hongyun Zhang, Shuopei Wang, Eryin Wang, Xiaobo Lu, Qian Li, Changhua Bao, Ke Deng, Haoxiong Zhang, Wei Yao, Guorui Chen, Alexei V. Fedorov, Jonathan D. Denlinger, Kenji Watanabe, Takashi Taniguchi, Guangyu Zhang, and Shuyun Zhou, "Experimental evidence of plasmarons and effective fine structure constant in electron-doped graphene/h-BN heterostructure," npj Quantum Materials , 1–7 (2021).

[67] Alireza Qaiumzadeh and Reza Asgari, "The effect of sublattice symmetry breaking on the electronic properties of doped graphene," New Journal of Physics **11**, 095023 (2009).

[68] Fabio Caruso and Feliciano Giustino, "Theory of electron-plasmon coupling in semiconductors," Physical Review B **94**, 1–7 (2016).

[69] S. Shallcross, S. Sharma, E. Kandelaki, and O. A. Pankratov, "Electronic structure of turbostratic graphene," Physical Review B - Condensed Matter and Materials Physics **81**, 165105 (2010).

[70] E. J. Mele, "Commensuration and interlayer coherence in twisted bilayer graphene," Physical Review B - Condensed Matter and Materials Physics **81**, 1–4 (2010).

[71] J. M.B. Lopes Dos Santos, N. M.R. Peres, and A. H. Castro Neto, "Continuum model of the twisted graphene bilayer," Physical Review B - Condensed Matter and Materials Physics **86**, 1–12 (2012).

[72] Zhao Dong Chu, Wen Yu He, and Lin He, "Coexistence of van Hove singularities and superlattice Dirac points in a slightly twisted graphene bilayer," Physical Review B - Condensed Matter and Materials Physics **87**, 2–9 (2013).

[73] Viktor Kandyba, Mikhail Yablonskikh, and Alexei Barinov, "Spectroscopic characterization of charge carrier anisotropic motion in twisted few-layer graphene," Scientific Reports **5**, 1–10 (2015).

[74] Elad Koren, Itai Leven, Emanuel Lörtscher, Armin Knoll, Oded Hod, and Urs Duerig, "Coherent commensurate electronic states at the interface between misoriented graphene layers," Nature Nanotechnology **11**, 752–757 (2016).

[75] S. Y. Zhou, G. H. Gweon, A. V. Fedorov, P. N. First, W. A. De Heer, D. H. Lee, F. Guinea, A. H. Castro Neto, and A. Lanzara, "Substrate-induced bandgap opening in epitaxial graphene," Nature Materials **6**, 770–775 (2007).

[76] B. Hunt, J. D. Sanchez-Yamagishi, A. F. Young, M. Yankowitz, B. J. LeRoy, K. Watanabe, T Taniguchi, P Moon, M Koshino, P. Jarillo-Herrero, and R C Ashoori, "Massive Dirac

Fermions and Hofstadter Butterfly in a van der Waals Heterostructure," Science **340**, 1427–1430 (2013).

[77] Gianluca Giovannetti, Petr A. Khomyakov, Geert Brocks, Paul J. Kelly, and Jeroen Van Den Brink, "Substrate-induced band gap in graphene on hexagonal boron nitride: Ab initio density functional calculations," Physical Review B - Condensed Matter and Materials Physics **76**, 2–5 (2007).

[78] Hakseong Kim, Nicolas Leconte, Bheema L. Chittari, Kenji Watanabe, Takashi Taniguchi, Allan H. Macdonald, Jeil Jung, and Suyong Jung, "Accurate Gap Determination in Monolayer and Bilayer Graphene/ h-BN Moiré Superlattices," Nano Letters **18**, 7732–7741 (2018).

[79] Nathan R. Finney, Matthew Yankowitz, Lithurshanaa Muraleetharan, K. Watanabe, T. Taniguchi, Cory R. Dean, and James Hone, "Tunable crystal symmetry in graphene–boron nitride heterostructures with coexisting moiré superlattices," Nature Nanotechnology **14**, 1029–1034 (2019).

[80] Jeil Jung, Ashley M. DaSilva, Allan H. MacDonald, and Shaffique Adam, "Origin of band gaps in graphene on hexagonal boron nitride," Nature Communications **6**, 6308 (2015).

[81] Valeri N. Kotov, Vitor M. Pereira, and Bruno Uchoa, "Polarization charge distribution in gapped graphene: Perturbation theory and exact diagonalization analysis," Physical Review B - Condensed Matter and Materials Physics **78**, 1–5 (2008).

[82] Yu Saito, Jingyuan Ge, Kenji Watanabe, Takashi Taniguchi, and Andrea F. Young, "Independent superconductors and correlated insulators in twisted bilayer graphene," Nature Physics **16**, 926–930 (2020).
